# The Use of Branching Agents in the Synthesis of PBAT

**DOI:** 10.3390/polym14091720

**Published:** 2022-04-22

**Authors:** Ilya E. Nifant’ev, Vladimir V. Bagrov, Pavel D. Komarov, Sergey O. Ilyin, Pavel V. Ivchenko

**Affiliations:** 1 A.V. Topchiev Institute of Petrochemical Synthesis RAS, 29 Leninsky Pr., 119991 Moscow, Russia; vlabag@yandex.ru (V.V.B.); komarrikov@yandex.ru (P.D.K.); s.o.ilyin@gmail.com (S.O.I.); or inpv@org.chem.msu.ru (P.V.I.); 2 Chemistry Department, M.V. Lomonosov Moscow State University, 1–3 Leninskie Gory, 119991 Moscow, Russia

**Keywords:** branching agents, long-chain branches, PBAT, polycondensation, rheometry

## Abstract

Biodegradable polyesters represent an advanced alternative to polyolefin plastics in various applications. Polybutylene adipate terephthalate (PBAT) can compete with polyolefins in terms of their mechanical characteristics and melt processing conditions. The properties of PBAT depend on the molecular weight, dispersity, and architecture of the copolymer. Long-chain branching (LCB) of the PBAT backbone is an efficient method for the improvement of the copolymer characteristics. In the present work, we studied branching agents (BAs) **1**–**7** of different structures in the two-stage polycondensation of 1,4-butanediol, dimethyl terephthalate, and adipic acid and investigated the composition and melt rheology of the copolymers. According to the results of the research, 1,1,1-tris(hydroxymethyl)ethane **2** and 3-hydroxy-2-(hydroxymethyl)-2-methylpropanoic acid **5** outperformed glycerol **1** as BAs in terms of shear thinning behavior and viscoelasticity.

## 1. Introduction

In recent years, the prospects of the greater use of the most commonly available petroleum-based plastics, polyolefins [1], have been limited by the high resistance of these materials to biodegradation. Therefore, despite their low cost and good mechanical characteristics, these materials are actually not considered as raw materials for disposable articles [2]. Biodegradable plastics, which are polyesters of different structures [3,4,5,6,7,8,9,10], represent an alternative to polyolefins. Most polyesters are far behind polyolefins in terms of mechanical properties. However, the product of the polycondensation of 1,4-butanediol, adipic acid (esters), and terephthalic acid (esters)—polybutylene adipate terephthalate (PBAT)—represents a synthetically available biodegradable [11] polyester that can compete with conventional polyolefins in terms of strength and elasticity [3,12,13,14,15].

**Scheme 1:**
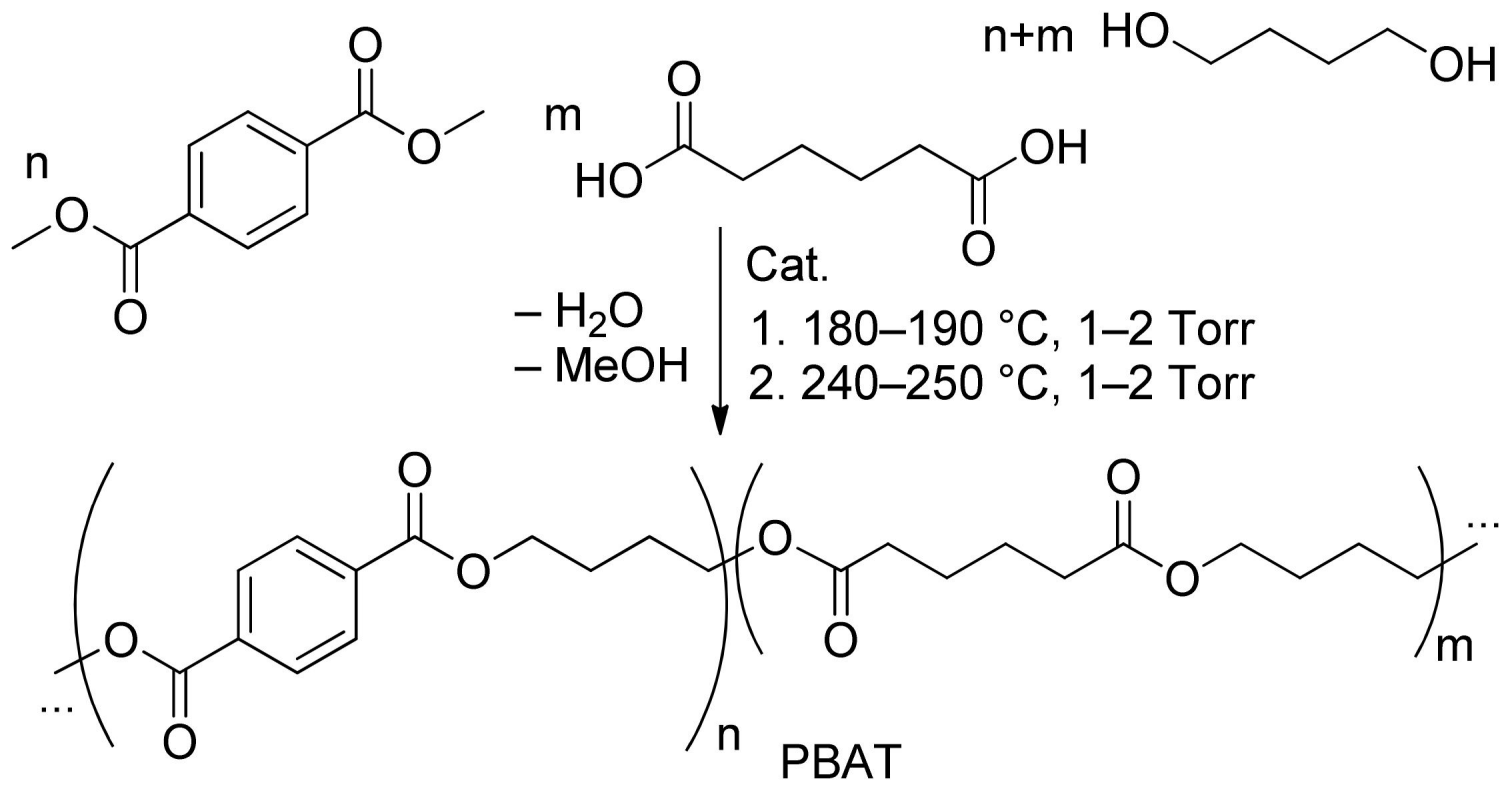
Synthesis of PBAT.

The synthesis of PBAT is based on the polycondensation of dimethyl terephthalate (DMT) and adipic acid or its esters with 1,4-butanediol. This reaction is usually conducted in two stages (Scheme 1), namely, a pre-polycondensation with the formation of relatively low-MW oligomers and a final polycondensation at elevated temperatures and under a high vacuum to reach target *M*_n_ values of ~50 kDa [15,16,17].

PBAT is currently regarded as a raw material or as a component of polymer composites that are designed to replace polyolefins in packaging and other fields of application [18,19,20,21,22,23,24,25]. Among these composites, PBAT blended with poly(*L*-lactide) (PLLA) and/or with starch has been studied intensively [26,27,28,29,30,31,32,33,34,35], but the poor miscibility of PBAT with these polymers on a molecular level makes it difficult to develop materials of the highest quality [31]. In packaging applications of both PBAT and PBAT-based composites, it is necessary to produce ultra-thin film using conventional technologies that were previously used in the polyolefin industry.

**Figure 1:**
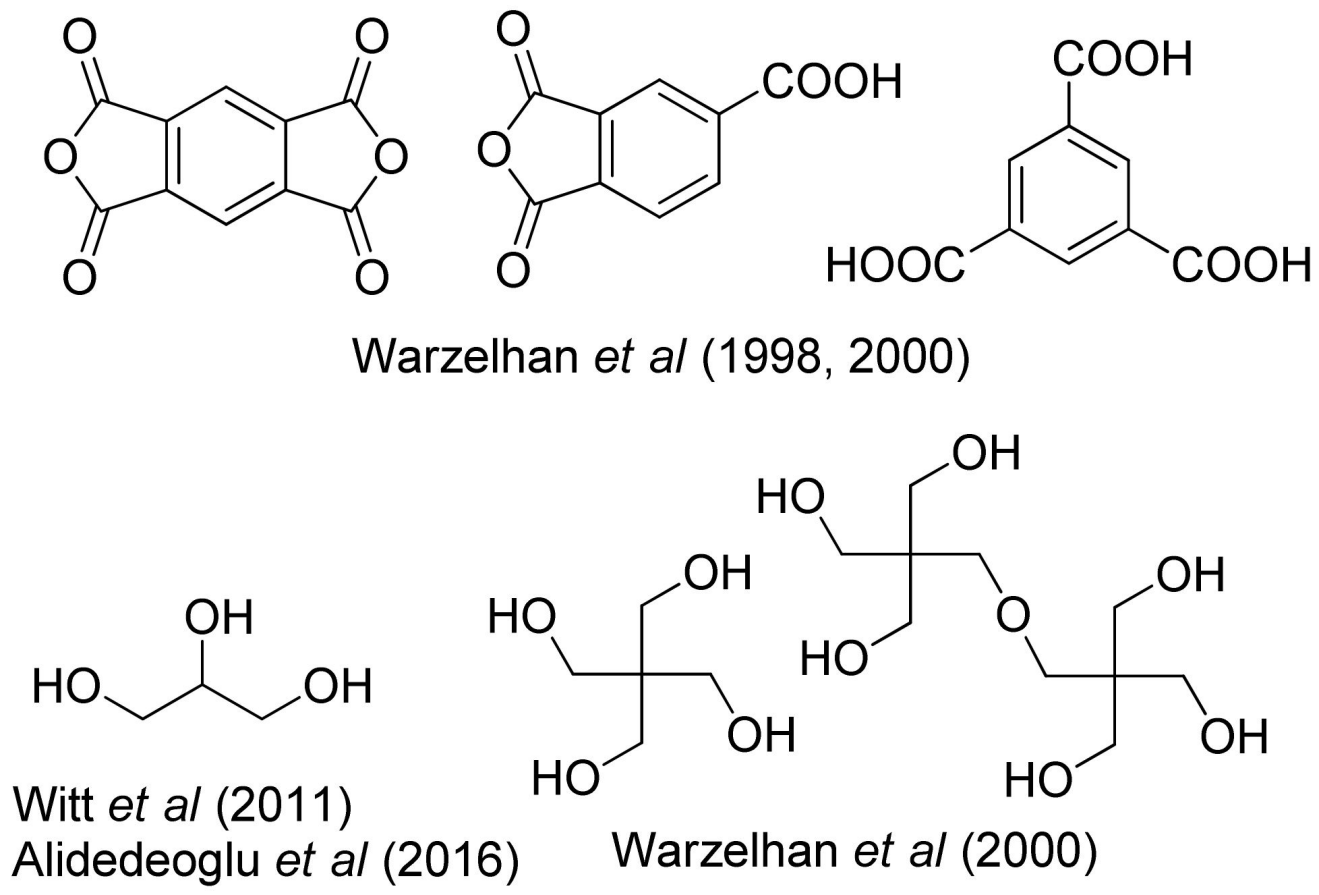
Branching agents used previously in PBAT synthesis; Warzelhan et al., (1998) [41], Warzelhan et al., (2000) [42,43], Witt et al., (2011) [44], Alidedeoglu et al., (2016) [45].

The most efficient way to reduce film thickness and enhance film strength is the improvement of the melt strength through the long-chain branching (LCB) of the polymer backbone; this approach was effectively used in the polyolefin industry [36,37,38,39,40]. The most common method of synthesizing LCB co-polyesters is to introduce multifunctional comonomers as branching agents (BAs) in polycondensation. For the synthesis of PBAT, only a few examples of the use of BAs have been reported in the patent literature [41,42,43,44,45] (Figure 1).

Surely, an alternative approach to the improvement of the mechanical characteristics of PBAT and PBAT blends is the use of ‘chain extenders’ at the stage of high-temperature molding and processing [46,47,48,49,50,51]. However, such a procedure results in the formation of cross-linked polymers with unpredictable characteristics and problematic recycling capabilities. It is no wonder, therefore, that the BASF company, the leading developer and manufacturer of PBAT (as the ECOFLEX^®^ polymer), uses glycerol as a BA in the modern continuous technology of the PBAT production, which is targeted toward obtaining LCB PBAT [44].

**Figure 2:**
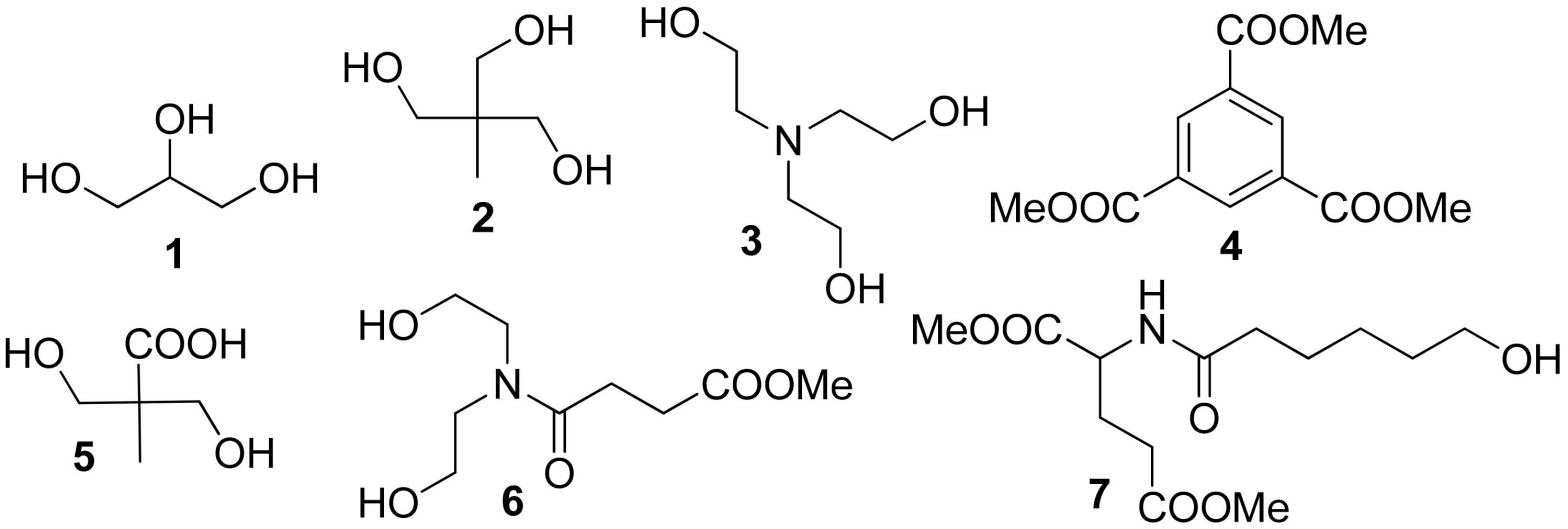
Branching agents **1**–**7** studied in this work.

In the present work, we tried to outline the promising path for the synthesis of LCB PBAT through a comparative study of the conventional BA glycerol **1** and a series of BAs **2**–**7** of different natures (Figure 2) in polycondensation. During the research, we synthesized a number of PBAT samples and investigated the microstructure and rheology of the copolymers with the aim of establishing the most efficient BAs.

## 2. Materials and Methods

### 2.1. Solvents and Reagents

Most of the solvents and chemicals were supplied by Merck (Darmstadt, Germany). Toluene (99.5%), triethylamine (≥99%), diethyl ether (Et_2_O, ≥99%), and tetrahydrofuran (THF, ≥99%) were refluxed over sodium/benzophenone ketyl and distilled. Ethanol (EtOH, ≥ 99%) was refluxed over Mg turnings (≥99.8%) and distilled. Diethanolamine, glutamic acid hydrochloride, ethyl acetate (EtOAc, ≥99%), DMT, 1,4-butanediol, adipic acid, glycerol, 1,1,1-tris(hydroxymethyl)ethane, triethanolamine, trimethyl 1,3,5-benzenetricarboxylate, 2,2-bis(hydroxymethyl)propionic acid, ε-caprolactone, dimethyl glutamate hydrochloride, and titanium (IV) butoxide (Ti(OBu)_4_) were used as purchased.

Methyl 4-chloro-4-oxobutanoate [52], 6-(benzyloxy)hexanoic acid [53], and 6-(benzyloxy)hexanoyl chloride [54] were synthesized according to the published procedures.

### 2.2. Analysis

The ^1^H and ^13^C {^1^H} NMR spectra were recorded on a Bruker AVANCE 400 spectrometer (400 MHz, Bruker Corporation, Billerica, MA, USA) at 20 °C. CDCl_3_ (D 99.8%, Cambridge Isotope Laboratories, Inc., Tewksbury, MA, USA) was used as purchased. Polymer solutions for the spectral study were prepared by using 1–2 h of dissolution of polymer samples (15–20 mg) in CDCl_3_ (0.7 mL) at 40 °C with stirring. The chemical shifts are reported relative to the solvent’s residual peaks (δ = 7.26 ppm for ^1^H and 77.16 for ^13^C {^1^H} NMR spectra).

Elemental analysis (C, H, N, O) was performed using a Perkin Elmer Series II CHNS/O Analyzer 2400 (Perkin Elmer, Waltham, MA, USA).

Size-exclusion chromatography (SEC) measurements were performed in THF (40 °C, flow rate: 1 mL/min) on a 1260 Infinity II (Agilent Technologies, Santa Clara, CA, USA) integrated instrument equipped with a PLgel MIXED-C column (2 × 10^2^–2 × 10^6^ Da), an autosampler, and a refractive index detector. The measurements were recorded with universal calibration according to a polystyrene standard.

The rheology of the polymer samples was examined with the Discovery HR-30 rotational rheometer (TA Instruments, New Castle, DE, USA) using a two-plate system with smooth surfaces (the diameter was 25 mm and the gap between the plates was 1 mm). Viscosity vs. temperature curves were obtained with a heating rate of 5 °C/min and a constant shear stress of 50 Pa in a temperature range of 150–200 °C. The dependences of the steady-state viscosity (*η*) and the first difference in normal stresses (*N*_1_) on the shear stress (σ) were determined by varying the shear rate (γ) in the range of 10^–3^–10^3^ s^–1^ at 190 °C. The frequency dependences of the loss tangent (tan*δ*) and complex viscosity (η*) were obtained in the linear viscoelasticity region by using the variation of the angular frequency (ω) from 0.0628 to 628 rad/s at 130 or 190 °C. The equations for calculating the rheological characteristics can be found elsewhere [55]; the relative error of their determination did not exceed 5%. The TRIOS 5.1.1 program package (TA Instruments, New Castle, DE, USA) was used for the treatment and presentation of the results.

### 2.3. Synthesis of Branching Agents ***6*** and ***7***

#### 2.3.1. Methyl 4-(bis(2-hydroxyethyl)amino)-4-oxobutanoate **6**

A solution of methyl 4-chloro-4-oxobutanoate (3.1 g, 20 mmol) [52] in THF (10 mL) was added dropwise at 0 °C with stirring to the mixture of diethanolamine (2.1 g, 20 mmol), triethylamine (2.4 mL, 20 mmol), and THF (50 mL). The mixture was allowed to warm to 20 °C; after 1 h of stirring, the mixture was filtered, the filtrate was evaporated under reduced pressure, and the residue was purified through column chromatography (EtOAc). The yield was 3.33 g (76%) of colorless oil. Elemental analysis: The following were calculated for C_9_H_17_NO_5_ (%): C, 49.31; H, 7.82; N, 6.39; O, 36.49; the following were found (%): C, 49.37; H, 7.84; N, 6.30; O, 36.49. ^1^H NMR (400 MHz, CDCl_3_, 20 °C) δ yielded: 4.23 (br, 2H); 3.75 (t, ^3^*J* = 5.2 Hz, 4H); 3.64 (s, 3H); 3.49 (q, ^3^*J* = 4.9 Hz, 4H); 2.70–2.60 (m, 4H). ^13^C {^1^H} NMR (101 MHz, CDCl_3_, 20 °C) δ yielded: 174.06; 173.40; 61.01; 60.62; 52.11; 51.94; 50.56; 29.26; 28.26. The NMR spectra of **6** are presented in Figures S1 and S2 in the Supplementary Materials.

#### 2.3.2. Methyl 4-(bis(2-hydroxyethyl)amino)-4-oxobutanoate **7**

Triethylamine (2.8 mL, 20 mmol) was added at 0 °C to the solution of glutamic acid hydrochloride (2.11 g, 10 mmol) in CH_2_Cl_2_ (40 mL). Then, a solution of 6-(benzyloxy)hexanoyl chloride (2.4 g, 10 mmol) [53,54] in CH_2_Cl_2_ (5 mL) was added. The mixture was allowed to warm to 20 °C; after 2 h of stirring, the mixture was washed with water, 5% HCl, 5% NaOH, water, and brine. The organic phase was dried over MgSO_4_ and evaporated under reduced pressure. The residue was dissolved in EtOAc (25 mL), Pd/C (10%, 300 mg) was added, and hydrogenation was conducted at 1 bar of H_2_ and 60 °C within 30 min. The catalyst was filtered off, the solvent was eliminated under reduced pressure, and the product was purified through column chromatography (EtOAc). The yield was 2.10 g (72%) of colorless oil. Elemental analysis: The following were calculated for C_13_H_23_NO_6_ (%): C, 53.97; H, 8.01; N, 4.84; O, 33.18; the following were found (%): C, 54.01; H, 8.04; N, 4.80; O, 33.15. ^1^H NMR (400 MHz, CDCl_3_, 20 °C) δ yielded: 6.59 (d, ^3^*J* = 7.8 Hz, 1H); 4.53 (m, 1H); 3.67 (s, 3H); 3.61 (s, 3H); 3.55 (t, ^3^*J* = 6.5 Hz, 2H); 2.73 (br, 1H); 2.34 (m, 2H); 2.18 (t, ^3^*J* = 7.4 Hz, 2H); 2.11 (m, 1H); 1.92 (m, 1H); 1.59 (m, 2H); 1.50 (m, 2H); 1.34 (m, 2H). ^13^C {^1^H} NMR (101 MHz, CDCl3 20 °C) δ yielded: 173.38; 173.33; 172.54; 62.24; 52.48; 51.86; 51.51; 36.14; 32.19; 30.11; 27.12; 25.26; 25.14. The NMR spectra of **7** are presented in Figures S3 and S4 in the Supplementary Materials.

### 2.4. Synthesis of Copolymers

Polycondensation experiments were conducted according to the modified version of a previously reported method [56]. DMT (56 g, 290 mmol), 1,4-butanediol (65 g, 720 mmol), and, optionally, BA (1.4 mmol) were placed into a round-bottom flask (250 mL) equipped with a condenser. Ti(OBu)_4_ (27 mg, 0.08 mmol) was added, and the mixture was heated to 150 °C. The condenser was replaced with a distillation head, and the heating was continued with the removal of methanol and THF. Adipic acid (52.98 g, 363 mmol) was added, and the reaction mixture was heated to 180 °C and stirred over an hour with the removal of water, methanol, and THF. Then, the flask was connected with a vacuum pump through a cold trap and heated to 180–190 °C at 1–2 Torr over 4 h. The flask was cooled, filled with argon, and equipped with a mechanical stirrer and short distillation head. Ti(OBu)_4_ (27 mg, 0.08 mmol) was added, and the mixture was heated to 240–250 °C at 1–2 Torr with stirring for 8 h. The hot copolymer melt was transferred into a PTFE cuvette, cooled, ground, and analyzed.

### 2.5. NMR Spectra of Copolymers

Despite decades of research on PBAT, only two publications have discussed the ^1^H NMR spectra of this copolymer in detail [57,58]. In the present study, we made some corrections to the signal assignment proposed in [57,58] and considered the presence of –(CH_2_)_3_CH_2_OH end-groups in the copolymers. The characteristic signals of these end-groups, which are attributed to chemical binding with terephthalate and adipate fragments (δ = 3.71 and 3.65 ppm, labels ‘f’ and ‘g’, respectively), are shown in the spectrum of the reaction mixture that was obtained at the first stage of the synthesis of PBAT without the addition of BAs (Figure S5 in the Supplementary Materials). This spectrum was used as a reference standard in our interpretation of the spectra of the PBAT samples obtained during our study. The details of the calculations of adipate/terephthalate (A/T) ratios are given in Section S2 in the Supplementary Materials, and the NMR spectra of the copolymers are presented in the Supplementary Materials in Figures S5–S12 (products of the 1st stage) and in Figures S13–S20 (products of the 2nd stage).

## 3. Results and Discussion

### 3.1. Selection and Synthesis of BAs

**Scheme 2:**
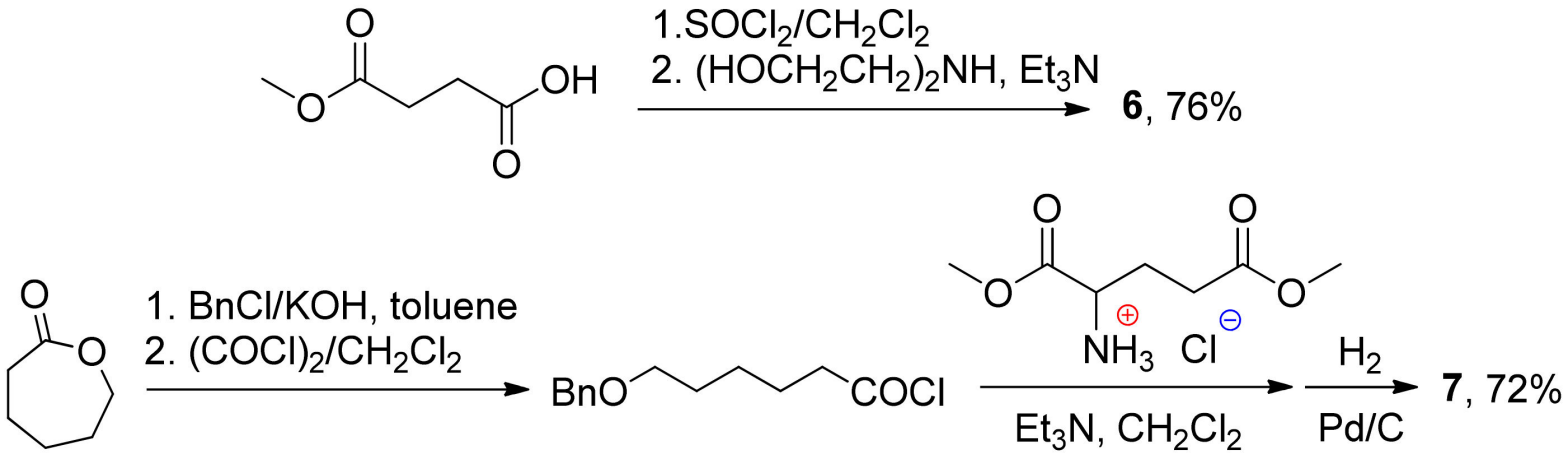
Synthesis of BAs **6** and **7**.

As can be seen in Figure 1, two different types of BAs were studied previously, namely, aromatic tri-/tetracarboxylic acids/anhydrides and polyols. In our study, we also used BAs of these two types (triols **1**–**3** and trimethyl ester **4**). However, since the reaction mixtures formed during the synthesis of PBAT contain both –CH_2_OH and –COOMe/–COOH reactive groups, we considered the molecules containing both aliphatic –COOMe/–COOH and –OH complementary functional groups **5**–**7** as prospective BAs. The 2,2-Bis(hydroxymethyl)propionic acid **5** is commercially available, and BAs **6** and **7** were synthesized from methyl succinate and ε-caprolactone/dimethyl glutamate, respectively (Scheme 2; for details, see Section 2.3; the NMR spectra of **6** and **7** are presented in Figures S1–S4 in the Supplementary Materials).

### 3.2. Synthesis of PBAT

#### 3.2.1. Polycondensation Experiments

As mentioned in Section 1, the synthesis of PBAT is usually conducted in two stages: at elevated temperatures (150–190 °C) with a formation of oligomers and at high temperatures (240–250 °C) to achieve target *M*_n_ values. The initial A/T molar ratio was 1.25; 0.2 mol% of BAs were added in all experiments, except for the synthesis of the linear copolymer (Table 1, Entries P1–P7 and P8). Early in the first stage, we carried out the reaction of DMT and the excess of 1,4-butanediol at 150 °C to obtain the corresponding diester as the main reaction product with the elimination of methanol and THF. This temperature was chosen to avoid the sublimation of DMT. Next, we added adipic acid and raised the reaction temperature to 180 °C at atmospheric pressure, and after 1 h, the pressure was reduced to 1–2 Torr. After 4 h at 180–190 °C, substantial parts of the reaction byproducts (water, THF, and traces of cyclic diester **8**; see Section 3.2.2) were distilled off. The products of the first stage represent relatively low-MW oligomers. As can be seen in Table 1, the A/T ratios in all reaction products remained virtually unchanged.

The second stage of the synthesis of PBAT was carried out at 240–250 °C under a vacuum (1–2 Torr) over 8 h. The reaction’s progress was provided by removing water at a high temperature in vacuo. During the second staged, we observed notable sublimation of the cyclic diester **8**; the yield of this byproduct (see Section 3.2.2) depended on the nature of the BAs (Table 1, Entries P1–P7).

The results of the experiments allowed us to draw conclusions about the use of the BAs of different natures. In particular, both glycerol **1** and 1,1,1-tris(hydroxymethyl)ethane **2** demonstrated comparable efficiency in the formation of relatively high-MW copolymers (Table 1, Entries P1 and P2, respectively). However, with the use of triethanolamine **3** as a BA, a relatively low-MW copolymer was obtained (Table 1, Entry P3). The MW of copolymer P4, which was obtained in the presence of triester **4**, was lower in comparison with the MW of the copolymers synthesized with the use of **1** and **2** (Table 1, Entry P4). This result is not so surprising when bearing in mind the lower reactivity of aromatic esters in polycondensation [17]. When BAs **5**–**7**, which included both aliphatic –COOH(Me) and –CH_2_OH functional groups, were used, high-MW copolymers were obtained (Table 1, Entries P5–P7), and the highest *M*_n_ value was detected by SEC for PBAT synthesized in the presence of **7**. When BAs were used effectively in terms of *M*_n_ values (Table 1, Entries P1, P2, P4–P7), the *Ð*_M_ values of the copolymers were substantially higher than the *Ð*_M_ values of the linear PBAT (Table 1, Entry P8)—2.15–2.40 vs. 1.95. A similar broadening of the MWD was previously observed for PBAT obtained with the use of BAs [15,41,42,43,44,45], which confirms the branched nature of the copolymers obtained.

#### 3.2.2. The Influence of the BAs on the Formation of Cyclic Diester **8** and on the A/T Ratio in PBAT

In most works devoted to the synthesis of PBAT [14,58,59,60,61], only water, MeOH, and THF were regarded as volatile reaction products. The formation of THF during polycondensation does not affect the A/T ratio in copolymers due to the use of excess 1,4-butanediol.

**Scheme 3:**
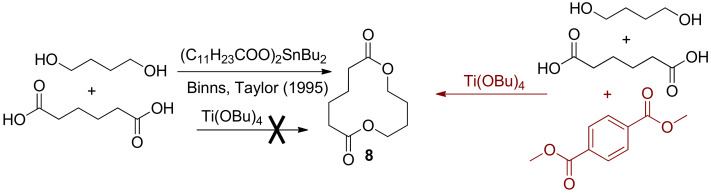
Preparation of 1,6-dioxacyclododecane-7,12-dione **8** through a tin (IV)-catalyzed reaction of adipic acid with 1,4-butanediol (Binns, Taylor (1995) [62]) and the formation of **8** during the synthesis of PBAT.

However, in our recent study [56], we detected the formation of an additional volatile reaction product, 1,6-dioxacyclododecane-7,12-dione **8** (Scheme 3; also see Figures S21 and S22 in the Supplementary Materials). Diester **8** was previously obtained through the reaction of adipic acid with 1,4-butanediol, catalyzed by dibutyltin dilaurate [62]. In the Ti(OBu)_4_ catalyzed reaction between adipic acid and 1,4-butanediol, we found no presence of **8** in considerable amounts, even at elevated temperatures; diester **8** was detected only after the addition of DMT. In our recent study [56], we showed that the formation of **8** is not strongly affected by the nature of the catalyst; the presence of phthalate is more important. This observation is awaiting a mechanistic explanation.

The formation of **8** at the second stage of the synthesis of PBAT is the cause of the change in the A/T ratio in high-MW copolymers in comparison with the products of the first stage. As a result, the yield of PBAT substantially decreased. As can be seen in Table 1, marked shifts in the A/T ratios were observed in all experiments except Entry P3 (low-MW copolymer). In terms of the copolymer yield (smaller changes in the A/T ratios), **1**, **2**, and **6** appear to be the preferred BAs.

### 3.3. Rheological Behavior of PBAT Polymers

Rheological experiments were conducted for the copolymer melts of P1, P2, and P4–P8; copolymer P3 was excluded from this study due to its low *M*_n_. Significant differences in *M*_n_ and comonomer compositions made it impractical to use the flow activation energies *E*_a_ as a criterion of branching, and more advanced and sensitive oscillation methods [63] were, therefore, used in follow-up studies.

#### 3.3.1. Shear-Thinning Behavior

**Figure 3:**
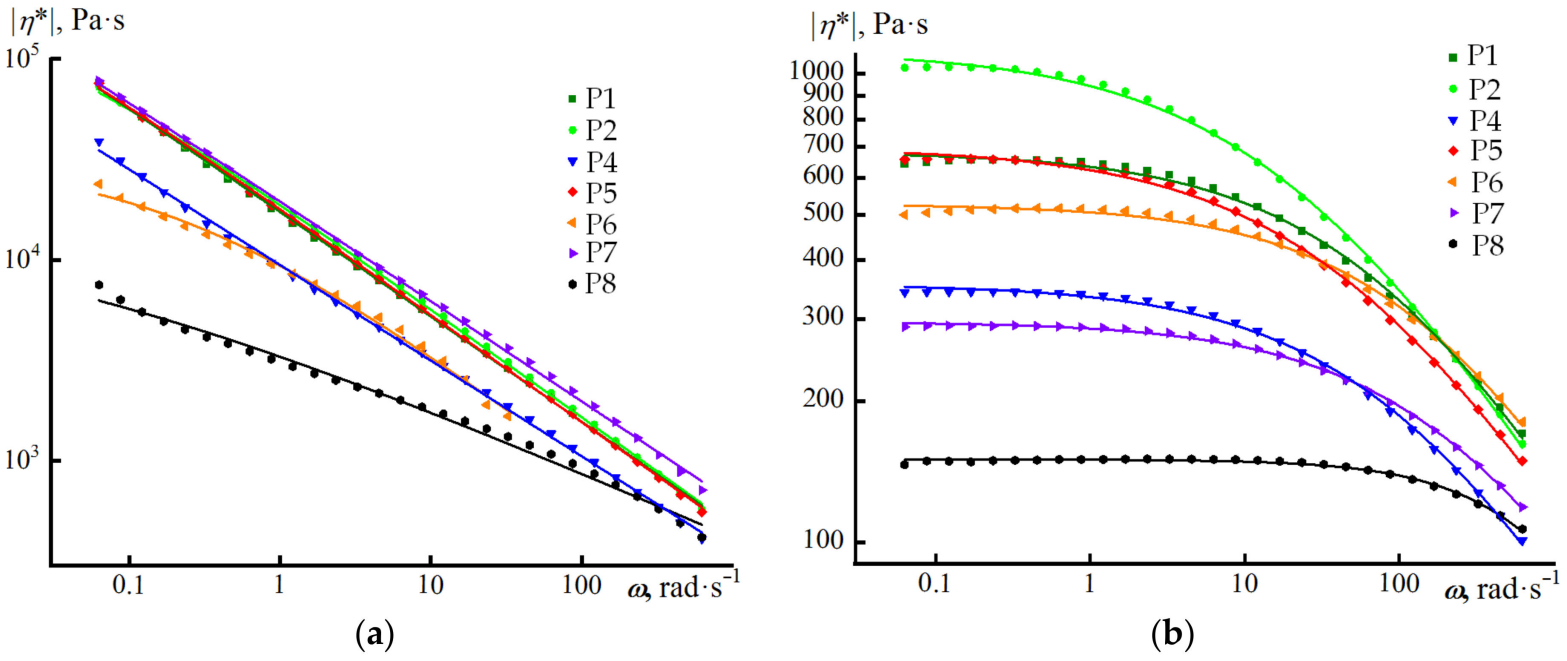
Frequency dependences of the complex viscosity at 130 (**a**) and 190 °C (**b**). The polymer numbers are presented in the legends. The dots show the experimental data, while the lines represent their fitting using the Cross equation.

The polymer melts exhibited shear-thinning behavior, which became more pronounced as they cooled (Figure 3). In this case, the samples differed both in the viscosity value and in the frequency at which the viscosity started to decrease. In general, the zero-shear viscosity of a polymer depends on its molecular weight *M* (Equation (1)).

(1) $$\eta =K_{\eta }M^{\alpha }$$

where *K_η_* is the fitting coefficient, while *α* is usually 3.4 for polymers and 1.0 for oligomers. Since the molecular weight was about the same for all samples (see Table 1), it would be reasonable to expect the same viscosity for them, which was not, in fact, the case.

The viscosity value can be affected by the width of the molecular weight distribution of the polymer *Ð*_M_ [64], which was approximately the same for all samples (Table 1). This means that the difference in the viscosity of copolymers P1–P8 (excluding P3) was dictated by the distinction in their branching. The point was that branching increased the glass transition temperature of the polymers and, therefore, their viscosity, as with a regular glass-forming liquid, according to the Williams–Landel–Ferry equation [65]. In addition, the frequency of the transition from Newtonian to non-Newtonian behavior was determined by the characteristic relaxation time of the polymer, which was likely also influenced by the branching of its chains. Therefore, the frequency dependences of the complex viscosity could be used to estimate the branching of the copolymers.

The frequency dependences of the complex viscosity can be approximated with the Cross equation (Equation (2)) [66].

(2) $$\eta =\eta_{\infty }+\frac{\eta_{0}-\eta_{\infty }}{1+{\left(\frac{\omega }{\omega_{0}}\right)}^{n}}$$

where *η*_∞_ is the high-shear viscosity, *η*_0_ is the zero-shear viscosity, *ω*_0_ is the nominal angular frequency (or shear rate) at which the system starts to exhibit non-Newtonian behavior, and *n* is the power-law index.

There were no regions of constant viscosity at low or high frequencies in the dependences obtained at 130 °C (Figure 3a), which meant that it was impossible to find *η*_∞_, *η*_0_, and *ω*_0_. Nevertheless, it was possible to estimate the power-law index (*n*, Table 2). The index took a small value for the linear polymer P8, whereas for the other samples, it lay in the range of 0.48–0.56.

An increase in the melt temperature up to 190 °C allowed the determination of *η*_0_ and *ω*_0_ (assuming *η*_∞_ = 0; see Table 2). In this case, the index took the highest value of 0.882 for the linear polymer P8, despite its non-Newtonian behavior, which was the least pronounced (Figure 3b). This meant that the power-law index was not suitable for evaluating the branching of the polymers, in contrast to the frequency of onset of non-Newtonian behavior (*ω*_0_, Table 2). The linear polymer P8 had the highest onset frequency, while the branched polymer P2 had the lowest one. Therefore, it could be assumed that its low *ω*_0_ also indicated that this sample had the most branched chains.

#### 3.3.2. Viscoelasticity

**Figure 4:**
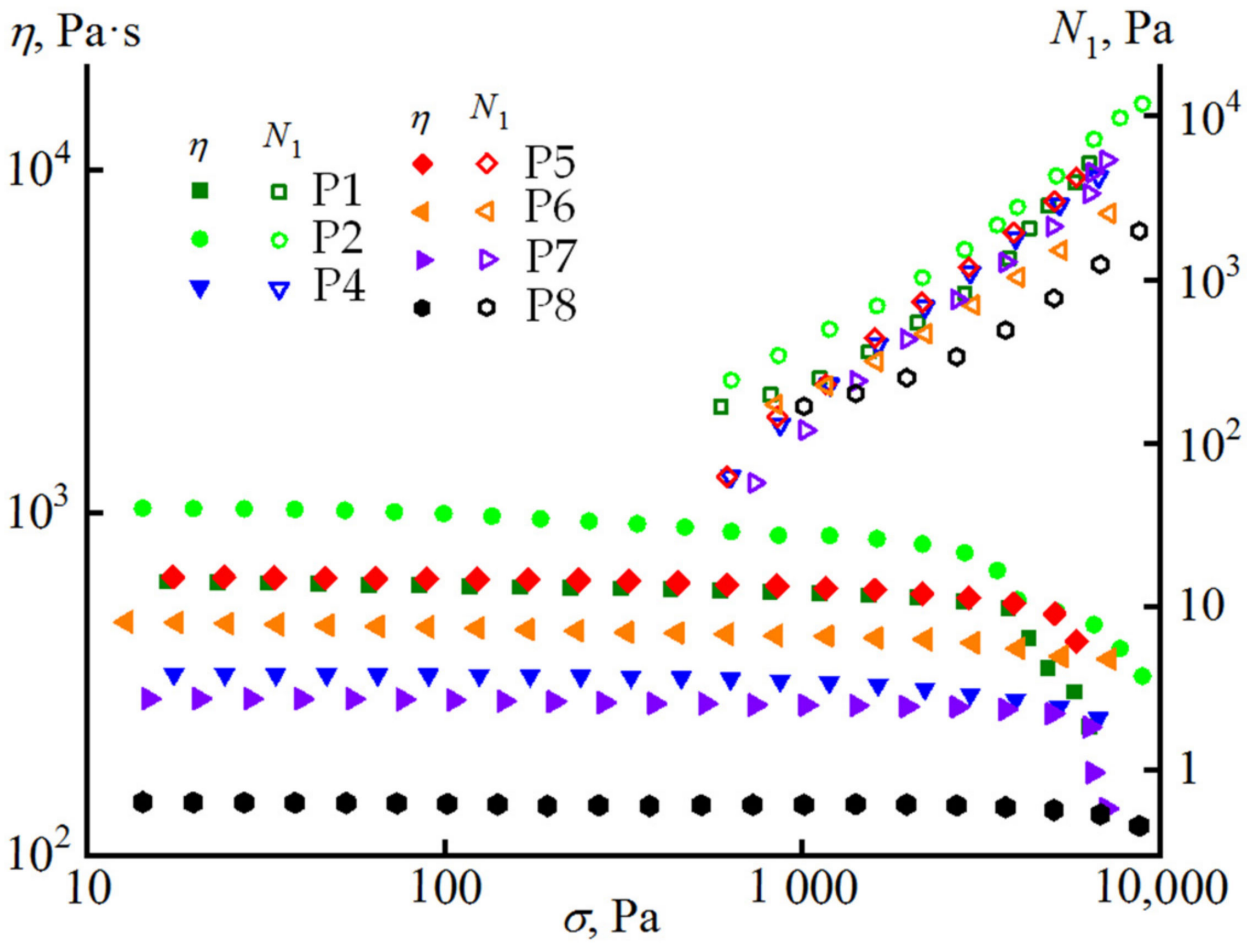
Shear stress dependences of the effective viscosity and the first normal stress difference at 190 °C. The polymer numbers are presented in the legend.

Thus, the greater chain branching contributed to the fact that the onset of the effective viscosity decrease occurred at lower angular frequencies or shear rates. However, not only the viscosity, but also the elasticity of the melt is important from the technological perspective of shaping polymer melts. Elasticity is important for suppressing the Plateau–Rayleigh instability of the polymer film during its formation and the possibility of its stretching with the orientation of macromolecular chains. The shaping process takes place at high deformation rates where non-Newtonian behavior is observed, and the point is that the decrease in effective viscosity is accompanied by an increase in melt elasticity. This was well detected experimentally when obtaining the flow curves of the polymers in the steady-state regime (Figure 4). At high shear rates or stresses, when the effective viscosity started to decrease, a normal reaction of the polymer melt arose. This was detected experimentally in the form of the first difference in normal stresses *N*_1_ [67]. In our case, polymer P2 has the highest *N*_1_ at the same shear stress, while the linear-chain sample P8 conversely had the lowest *N*_1_. This also pointed in favor of the greatest branching of specimen P2.

**Figure 5:**
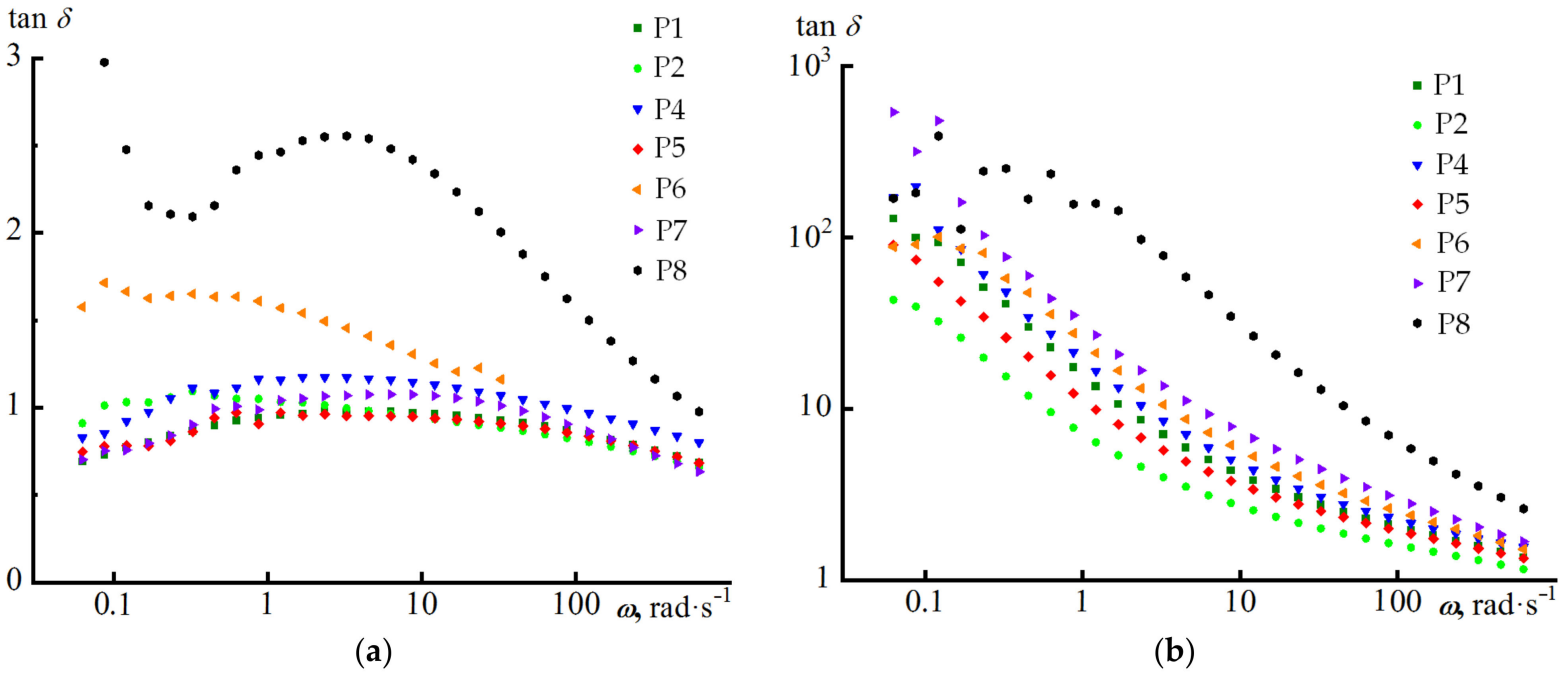
Frequency dependences of the loss tangent at 130 (**a**) and 190 °C (**b**). The polymer numbers are presented in the legends.

The ability to exhibit an elastic response during deformation could also be evaluated using the small-amplitude oscillatory shear technique in terms of measuring the dependences of the storage and loss moduli (Figure S32 in the Supplementary Materials). The ratio of the storage modulus to the loss modulus gave the value of the loss tangent. The smaller the loss tangent is, the higher the elastic response will be, which becomes dominant when tan*δ* < 1. When considering the frequency dependences of the loss tangent at 130 °C (Figure 5a), it turned out that only the non-branched sample P8 always had tan*δ* > 1, while for other polymers, tan*δ* ≈ 1. The latter meant that our branched polymers could not be formed at 130 °C because of their prevailing elasticity, which could lead to various instabilities [68]. However, the linear polymer P8 could not be formed anyway due to its excessively high viscosity at this temperature (the melt viscosity should be about 1000 Pa·s; see Figure 3a).

At the higher temperature of 190 °C, the situation changed, and tanδ became <1 for all polymers (Figure 5b). Now, of greater importance from the viewpoint of shaping technology is the tendency of the loss tangent toward 1.0 as the angular frequency increases. Polymer P2 had the lowest loss tangent in the entire frequency range, which was evidence in favor of its greater branching compared to that of other polymers of comparable molecular weight.

Thus, the shaping requires a balance between the elasticity and viscosity of the melt, and the Weissenberg number serves as such a criterion that characterizes the ratio of elastic forces to viscous ones (Equation (3)) [69].

(3) $$Wi=\frac{N_{1}}{\sigma }$$

It is essential that the Weissenberg number be less than 1 at extrusion speeds, while the opposite should be true at higher rates of film elongation. Meanwhile, the Weissenberg number can easily be lowered by increasing the temperature, as this will reduce the viscosity and not cause problems in the case of a heat-resistant polymer. The problem may be to increase the Weissenberg number, because lowering the temperature may not be technically realizable due to a significant increase in melt viscosity. For this reason, it is important to synthesize a polymer that provides the highest Weissenberg number at a given temperature.

**Figure 6:**
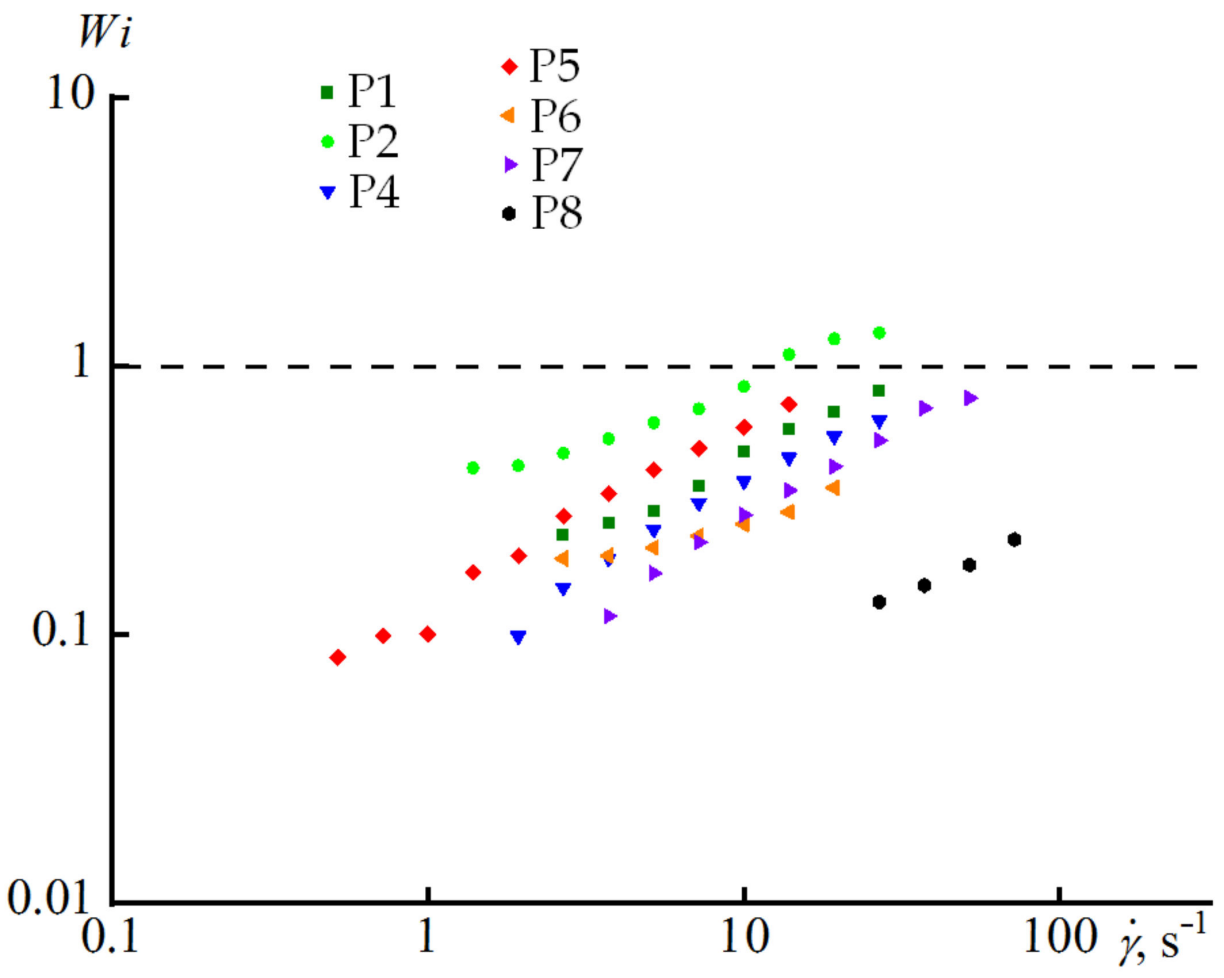
Shear rate dependences of the Weissenberg number at 190 °C. The polymer numbers are presented in the legends.

In our case, polymer P2 outperformed all other samples, as it had the highest Weissenberg number at a certain temperature and shear rate (Figure 6). However, the viscosity was also important, which was higher in the case of sample P2 (Figure 3b and Figure 4), making it more difficult to process at first glance.

**Figure 7:**
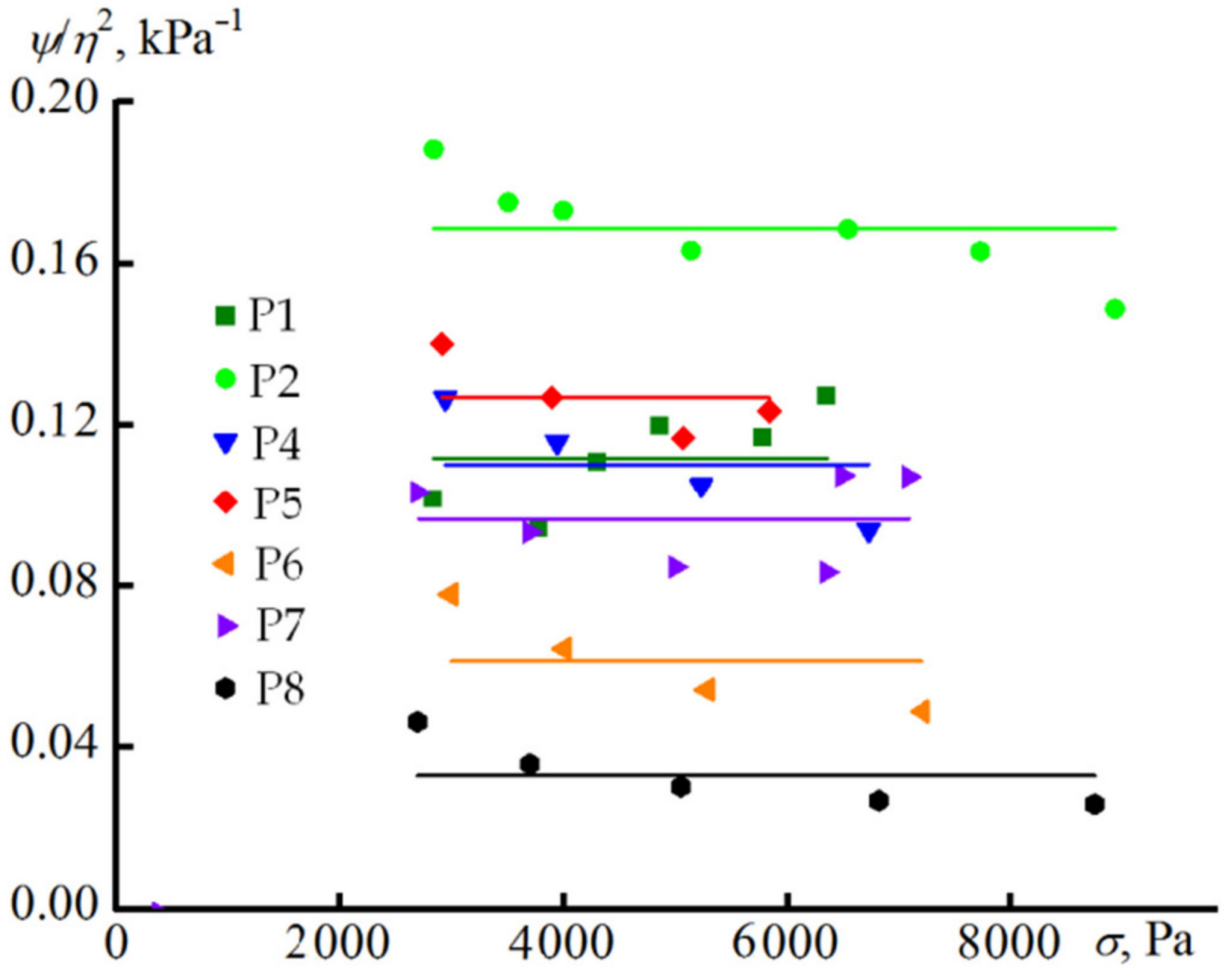
Shear stress dependence of the ratio between the coefficient of the first normal stress difference and the square of the viscosity at 190 °C. The polymer numbers are presented in the legends.

The elasticity of the polymer expressed through the coefficient of the first normal stress difference (a measure of elasticity similar to viscosity (Equation (4)) is determined by its molecular weight (Equation (5)) [64].

(4) $$\psi =\frac{N_{1}}{\gamma^{2}}$$

(5) $$\psi =K_{\psi }M^{2\alpha }$$

where *K_ψ_* is the fitting coefficient. It is easy to see that Equations (1) and (5) are very similar. Moreover, the ratio between *ψ* and *η*^2^, which characterizes the ability of macromolecules to undergo reversible deformations, should not depend on the polymer’s molecular weight: *ψ*/*η*^2^ = *const*. Meanwhile, it turned out that *ψ*/*η*^2^ was very different for our polymers (Figure 7). This meant that they differed in the architecture of their chains. The *ψ*/*η*^2^ ratio was the highest for polymer P2, indicating its greater melt strength at the same viscosity. In turn, this indicated that the branching of this polymer was the highest in comparison to all other samples.

## 4. Conclusions

**Figure 8:**
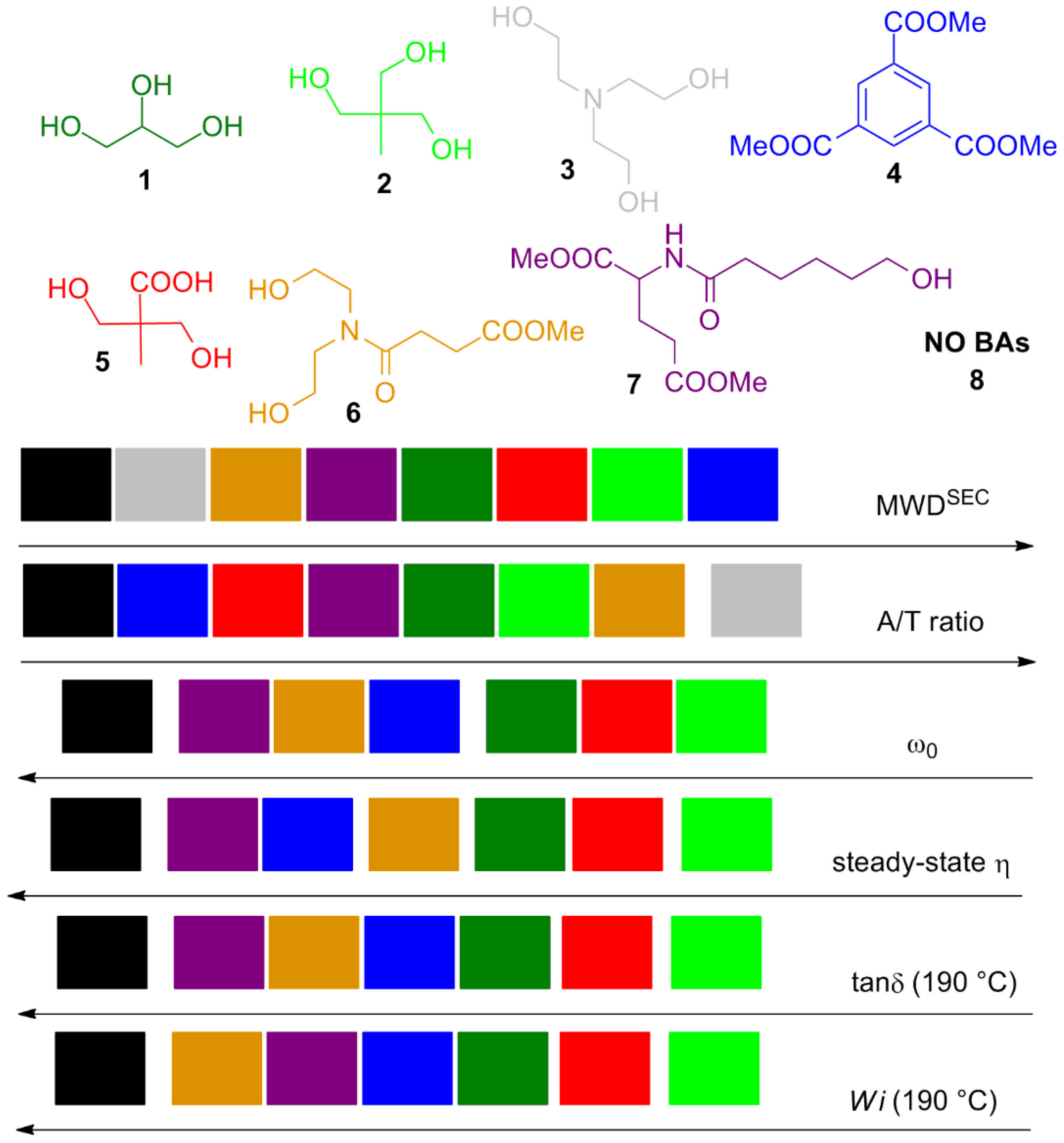
The main characteristics of the PBAT synthesized.

Long-chain branched PBATs P1–P7 were successfully synthesized via a direct polycondensation in the presence of Ti(OBu)_4_ by using branching agents (BAs) **1**–**7** of different chemical structures (Figure 2). Branched copolymers P1–P7 were studied along with a benchmark linear sample P8 through NMR spectroscopy, SEC, and precision rheometry. The study led to the following generalized conclusions (Figure 8):

- The use of triethanolamine **3** as a BA resulted in the formation of a low-MW copolymer with a qualitatively different microstructure (with the absence of –CH_2_OH end-groups; see Figure S15 in the Supplementary Materials).
- The values of *Ð*_M_ (SEC) of copolymers P1, P2, and P4–P7, which were obtained in the presence of BAs, were higher than the *Ð*_M_ value of the linear copolymer P8.
- The A/T ratios and yields of the polycondensation products were higher for branched copolymers P1–P7.
- The frequency of onset of non-Newtonian behavior *ω*_0_, which was inversely proportional to the level of branching, had the maximum value for P8 and the minimum value for P2. According to this criterion, branched polymers may be divided into two groups—relatively high-branched polymers (P2, P5, P1) and low-branched polymers (P6, P7). Sample P4 had a transitory position.
- The steady-state flow curves, tan*δ* curves, and shear rate dependences of the Weissenberg number show similar patterns.

In this way, 1,1,1-tris(hydroxymethyl)ethane **2** represents an excellent branching agent for the synthesis of PBAT, and 2,2-bis(hydroxymethyl)propionic acid **5** also demonstrated promising characteristics. As can be seen in Figure 8, these BAs outperform the conventional BA glycerol **1** possible due to the higher thermal stability of **2** and **5**. We believe that further studies on the design and testing of BAs at the polycondensation stage are essential for the development of advanced polyesters and polyester-based composites.

## Figures and Tables

**Table 1:** Synthesis and characteristics of PBAT (first stage: 150–190 °C, 760 to 1–2 Torr; second stage: 240–250 °C, 1–2 Torr).

Entry	BA	Product of the First Stage			Product of the Second Stage				
		A/T ratio ^1^	DP_n_^NMR 2^	*M*_n_^NMR^, kDa ^3^	A/T ratio	DP_n_^NMR^	*M*_n_^NMR^, kDa	*M*_n_^SEC^, kDa	*Ð*_M_ ^4^
P1	**1**	1.25	5.61	1.32	1.15	71.58	15.1	45.2	2.26
P2	**2**	1.25	6.51	1.51	1.16	71.92	15.2	46.4	2.40
P3	**3**	1.24	8.15	1.85	1.24	–	–	18.0	2.08
P4	**4**	1.25	5.10	1.21	1.11	60.36	12.8	37.2	2.42
P5	**5**	1.24	6.69	1.54	1.11	60.14	12.8	43.8	2.37
P6	**6**	1.23	7.09	1.63	1.18	62.21	13.2	47.1	2.15
P7	**7**	1.22	8.37	1.90	1.12	84.70	17.9	52.6	2.15
P8	no	1.23	7.77	1.42	1.06	68.58	14.5	41.3	1.95

^1^ The molar ratio of adipate and terephthalate fragments. ^2^ Degree of polymerization, ^1^H NMR data. ^3^ Number average molecular weight, NMR data. ^4^ Dispersity, SEC data.

**Table 2:** Rheological characteristics of PBAT.

Entry	130 °C	190 °C			*ψ*/*η*^2^, Pa^−1 4^
	*n* ^1^	*η*_0_, Pa·s ^2^	*ω*_0_, s^−1 3^	*n*	
P1	0.524	681	88.5	0.574	0.112 ± 0.005
P2	0.540	1120	22.3	0.538	0.169 ± 0.005
P4	0.476	356	118.5	0.573	0.110 ± 0.007
P5	0.535	696	53.5	0.537	0.127 ± 0.005
P6	0.560	527	202.4	0.601	0.061 ± 0.006
P7	0.500	296	311.5	0.586	0.096 ± 0.005
P8	0.330	150	1670	0.882	0.033 ± 0.004

^1^ Power-law index. ^2^ Zero-shear viscosity. ^3^ Nominal angular frequency of non-Newtonian behaviour. ^4^ The ratio of elasticity and viscosity squared.

## Data Availability

The data presented in this study are available on request from the corresponding author.

## Supplementary Materials

The following are available online at https://www.mdpi.com/article/10.3390/polym14091720/s1, Figure S1: ^1^H NMR spectrum (400 MHz, CDCl_3_, 20 °C) of **6**, Figure S2: ^13^C {^1^H} NMR spectrum (101 MHz, CDCl_3_, 20 °C) of **6**, Figure S3: ^1^H NMR spectrum (400 MHz, CDCl_3_, 20 °C) of **7**, Figure S4: ^13^C {^1^H} NMR spectrum (101 MHz, CDCl_3_, 20 °C) of **7**, Figures S5–S11: ^1^H NMR spectra (400 MHz, CDCl_3_, 20 °C) of PBAT oligomers obtained with the use of **1**–**7**, respectively, Figure S12: ^1^H NMR spectrum (400 MHz, CDCl_3_, 20 °C) of the PBAT oligomer obtained in the absence of BAs, Figures S13–S20: ^1^H NMR spectra (400 MHz, CDCl_3_, 20 °C) of PBAT samples P1–P8, respectively, Figure S21: ^1^H NMR spectrum (400 MHz, CDCl_3_, 20 °C) of **8**, Figure S22: ^13^C {^1^H} NMR spectrum (101 MHz, CDCl_3_, 20 °C) of **8**, Figures S23–S30: DSC plots of PBAT samples P1–P8, respectively, Figure S31: Dependence of the glass transition temperature of PBAT on the content of butylene adipate units. The dots show experimental data, while the line represents the result of a theoretical calculation using the Fox equation. The polymer numbers (see Table S1) are indicated near the dots. The data for low-MW copolymer P3 are not presented, Figure S32: Frequency dependences of the storage and loss moduli at 130 (left) and 190 °C (right). The polymer numbers are presented in the legends. References [50,56,62,70,71,72,73,74,75] are cited in the Supplementary Materials.
