# Supplementary material for: The Use of Branching Agents in the Synthesis of PBAT
Source: Polymers (Basel). 2022 Apr 22;14(9):1720. doi: 10.3390/polym14091720 (PMC9100140; doi:10.3390/polym14091720)
Supplement: Supplementary file 1 [file polymers-14-01720-s001.zip › polymers-1656489-supplementary.pdf]

polymers

MDPI

Article

The Use of Branching Agents in the Synthesis of PBAT

Ilya E. Nifant'ev [1,2,*], Vladimir V. Bagrov [1,2], Pavel D. Komarov [1], Sergey O. Ilyin [1] and Pavel V. Ivchenko [1,2]

[1] A.V. Topchiev Institute of Petrochemical Synthesis RAS, 29 Leninsky Pr., 119991 Moscow, Russia; vlabag@yandex.ru (V.V.B.); komarrikov@yandex.ru (P.D.K.); s.o.ilyin@gmail.com (S.O.I.); phpasha1@yandex.ru or inpv@org.chem.msu.ru (P.V.I.).

[2] Chemistry Department, M.V. Lomonosov Moscow State University, 1–3 Leninskie Gory, 119991 Moscow, Russia;

* Correspondence: ilnif@yahoo.com or inif@org.chem.msu.ru

Supplementary Information

S1. ^{1}H and ^{13}C NMR spectra of **6** and **7** S2–S3
S2. ^{1}H NMR spectra of polycondensation products S4–S12
    S2.1. ^{1}H NMR spectra of the PBAT oligomers (1st stage) S4–S7
    S2.2. ^{1}H NMR spectra of the PBAT (2nd stage) S8–S11
    S2.3. ^{1}H and ^{13}C NMR spectra of **8** S12
S3. Thermal properties of PBATs S13
S4. Rheology of PBATs S18
References S19

S1. ^{1}H and ^{13}C NMR spectra of 6 and 7

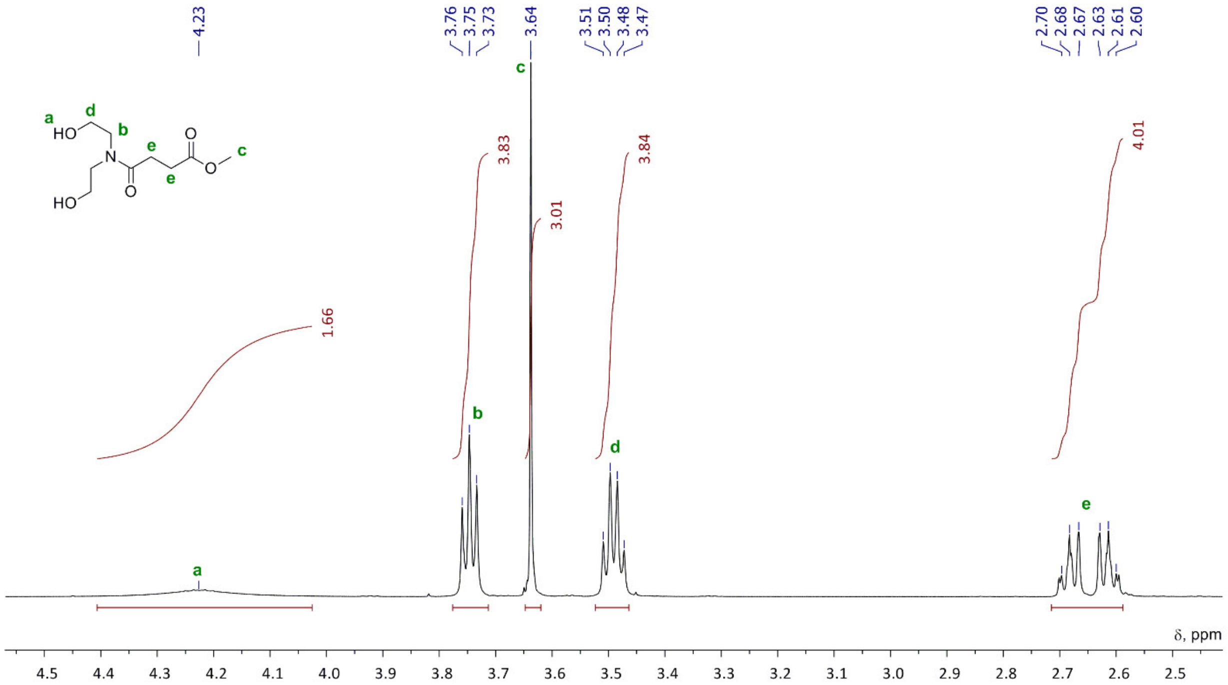

Figure S1. ^{1}H NMR spectrum (400 MHz, $CDCl_3$, 20 °C) of **6**.

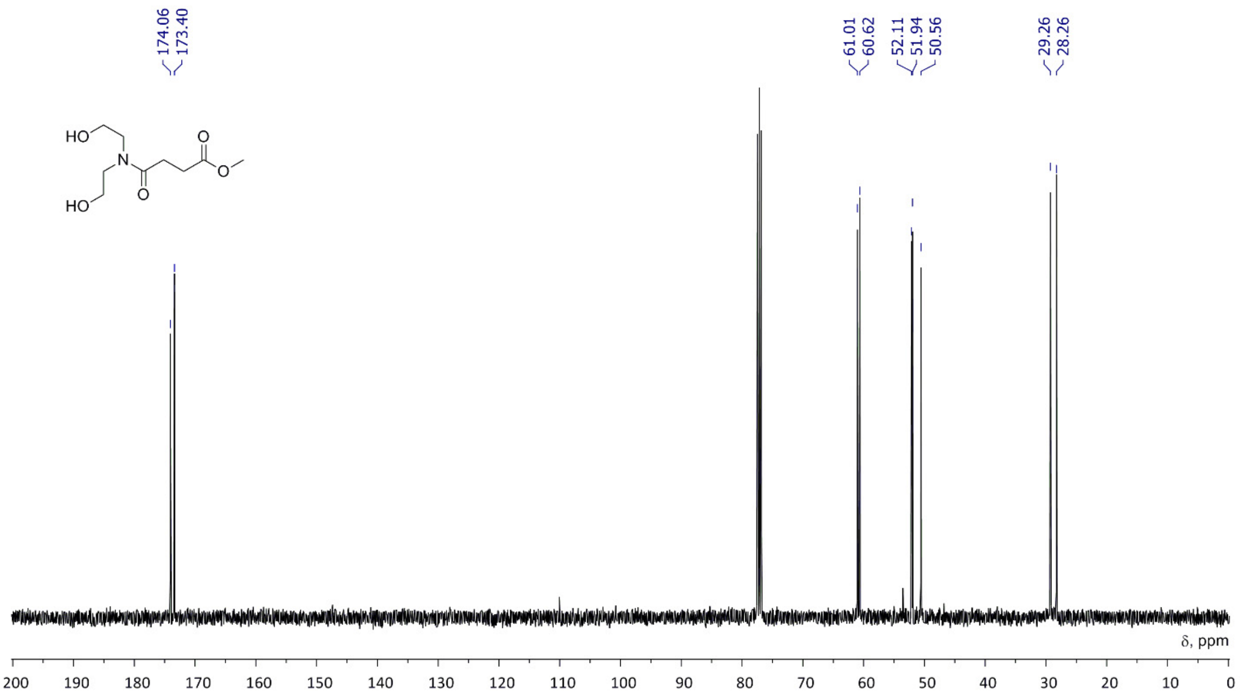

Figure S2. ^{13}C {^{1}H} NMR spectrum (101 MHz, $CDCl_3$, 20 °C) of **6**.

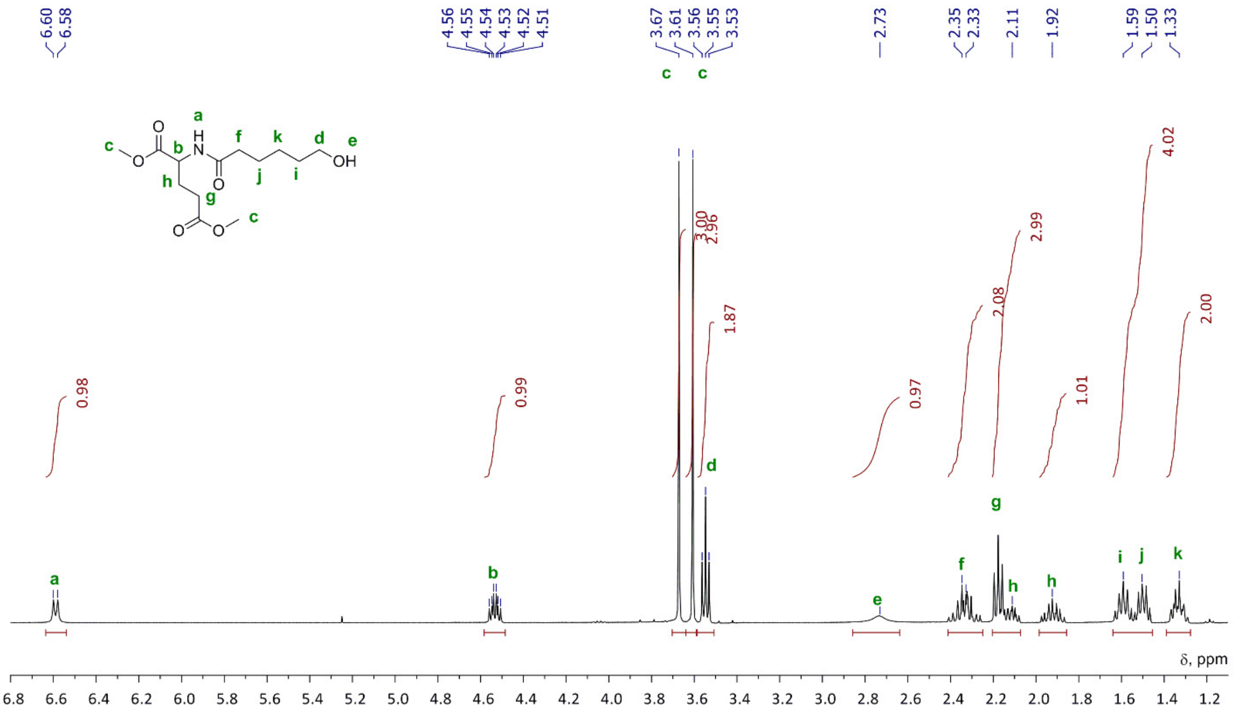

Figure S3. ^{1}H NMR spectrum (400 MHz, $CDCl_3$, 20 °C) of **7**.

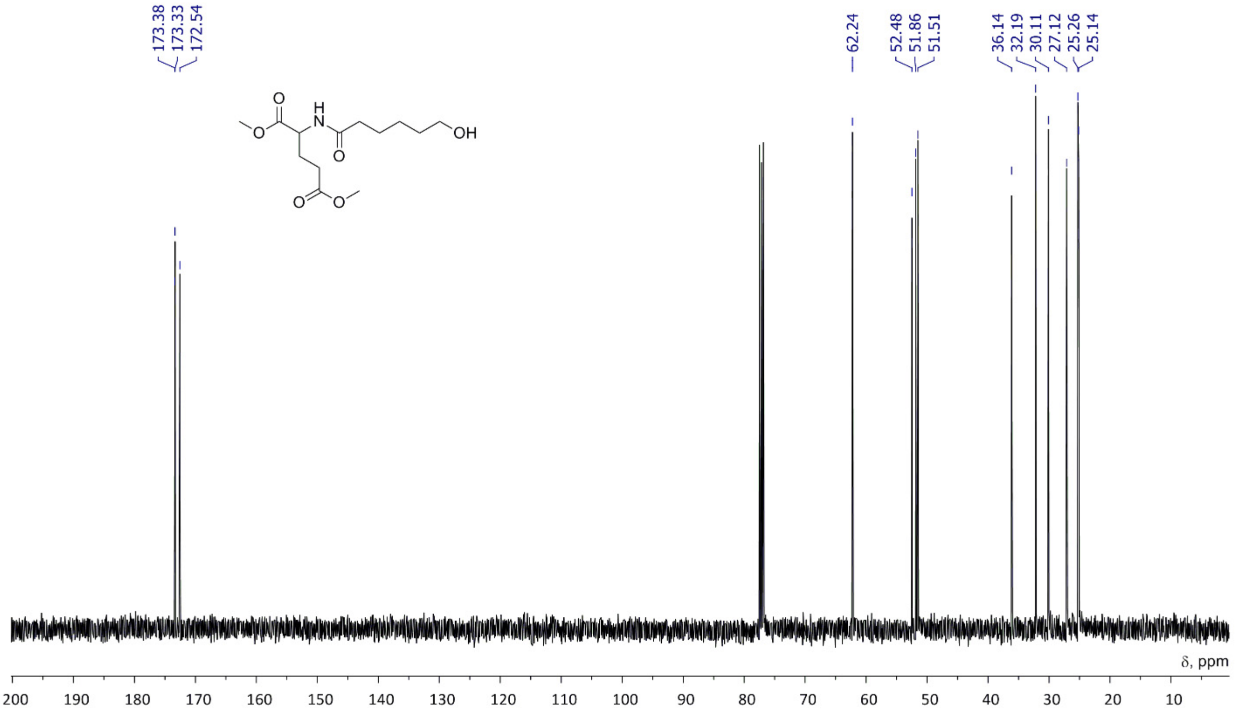

Figure S4. ^{13}C {^{1}H} NMR spectrum (101 MHz, $CDCl_3$, 20 °C) of **7**.

S2. NMR spectra of polycondensation products

S2.1. ¹H NMR spectra of the PBAT oligomers (1st stage)

¹H NMR spectrum of the product of the 1st stage of polycondensation is presented in Figure S5.

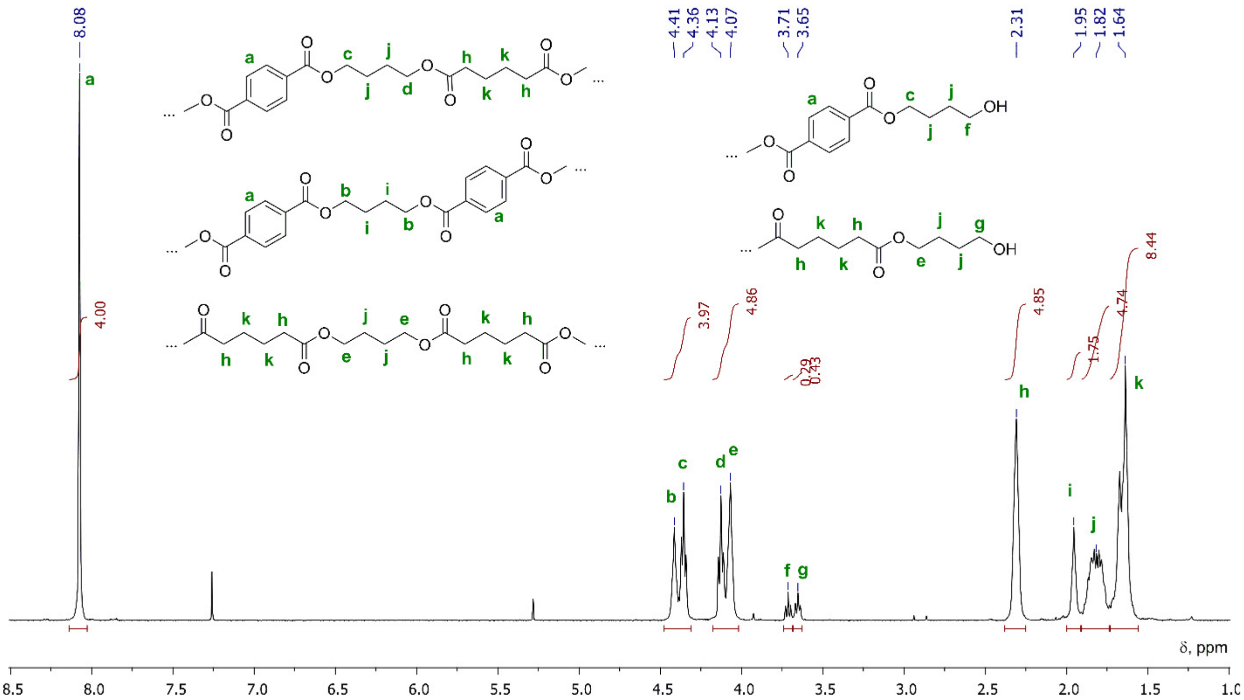

Figure S5. ¹H NMR spectrum (400 MHz, $CDCl_3$, 20 °C) of the product of the first stage of PBAT synthesis obtained in the absence of BAs.

Adipate/terephthalate (A/T) ratios of the oligomers were calculated based on the integral intensities of terephthalate (δ = 8.08 ppm) and α-CH_2 adipate (δ = 2.31 ppm) signals in ¹H NMR spectra (Figure S5, labels 'a' and 'h', respectively). The values of the degree of polymerization (DP_n) and M_n^{NMR} were calculated using Equations S1 and S2, respectively

$$DP_n = \frac{IE(\mathrm{a}) + IE(\mathrm{h})}{2 \cdot (IE(\mathrm{f}) + IE(\mathrm{g}))}, \quad \text{(S1)}$$

$$M_n^{NMR} = DP_n \cdot \left(\frac{148.12 \cdot IE(\mathrm{a}) + 138.13 \cdot IE(\mathrm{h})}{IE(\mathrm{a}) + IE(\mathrm{h})} + 72.11\right) + 2 \cdot 73.11, \quad \text{(S2)}$$

where *IE*(a), *IE*(f), *IE*(g) and *IE*(h) are integral intensities of the corresponding signals in ¹H NMR spectra (see Figure S5); 148.12, 138.13 and 72.11 are molecular weights (Da) of terephthalate, adipate and 1,4-butanediol derived fragments in PBAT. Note that Equations S1 and S2 are based on the assumption that PBAT macromolecule contains two –$(CH_2)_4OH$ end fragments (MW 73.11 Da) due to excess of 1,4-butanediol in the reaction mixture.

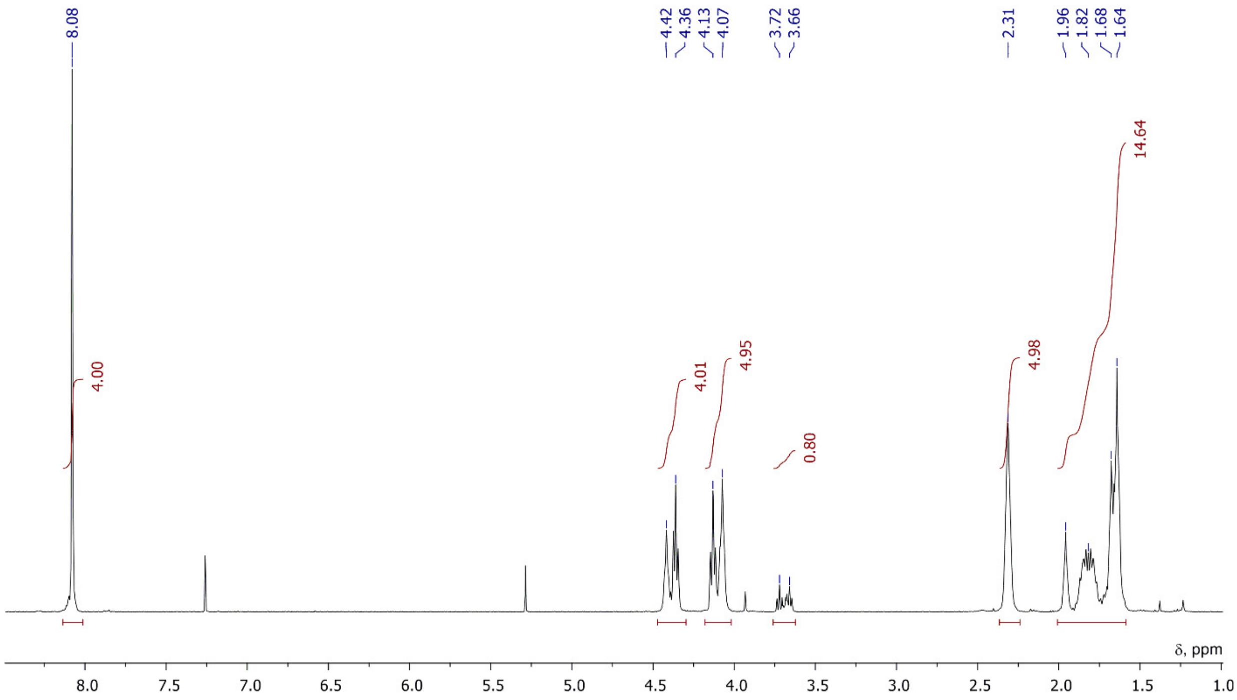

Figure S6. ^{1}H NMR spectrum (400 MHz, $CDCl_3$, 20 °C) of PBAT oligomer, obtained with the use of **1**.

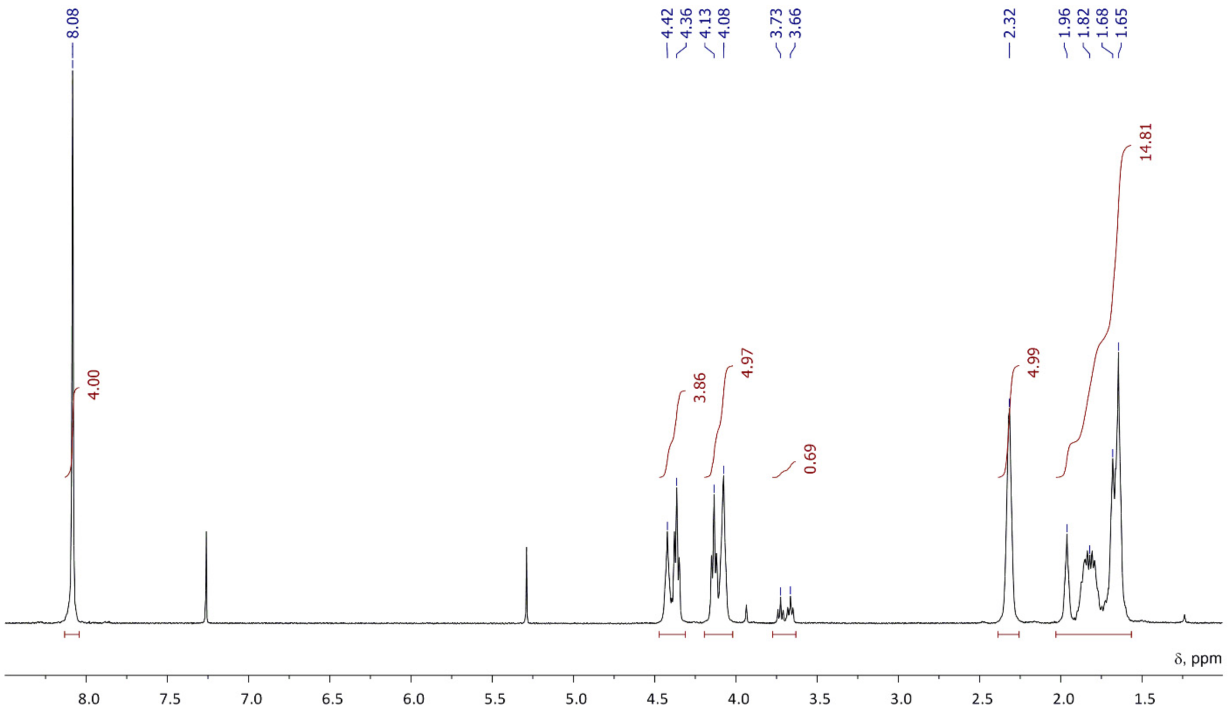

Figure S7. ^{1}H NMR spectrum (400 MHz, $CDCl_3$, 20 °C) of PBAT oligomer, obtained with the use of **2**.

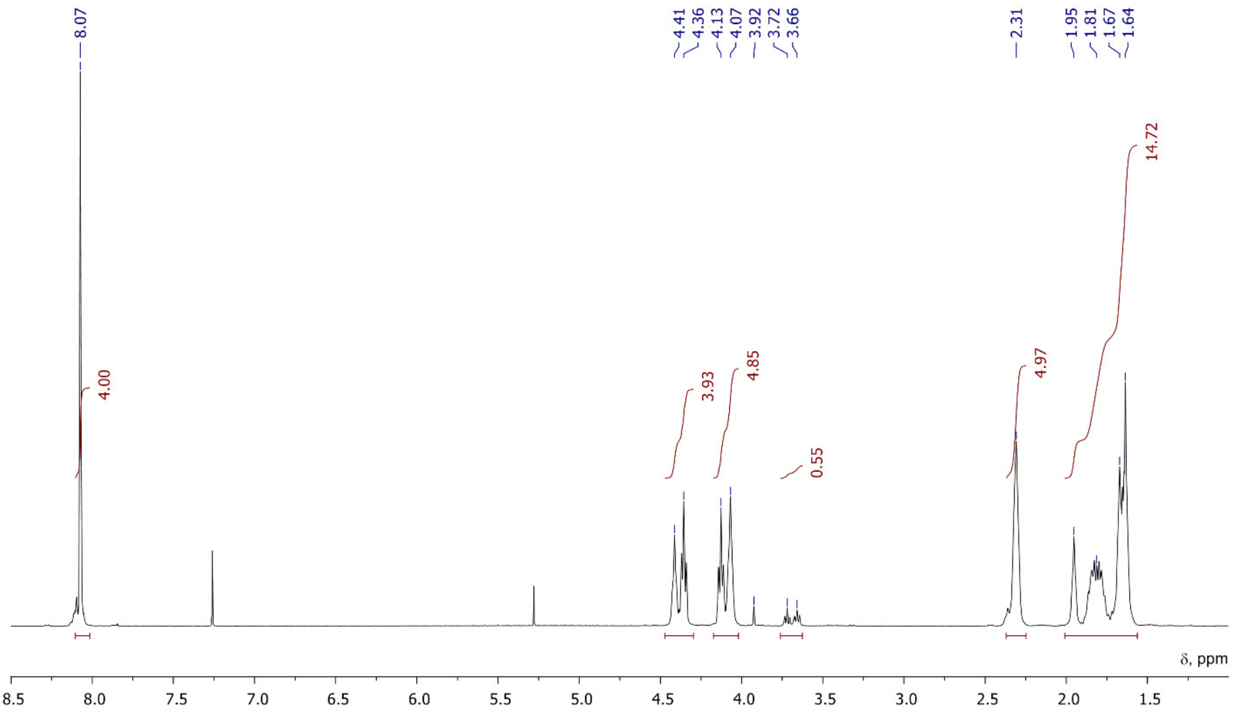

Figure S8. ^{1}H NMR spectrum (400 MHz, $CDCl_3$, 20 °C) of PBAT oligomer, obtained with the use of **3**.

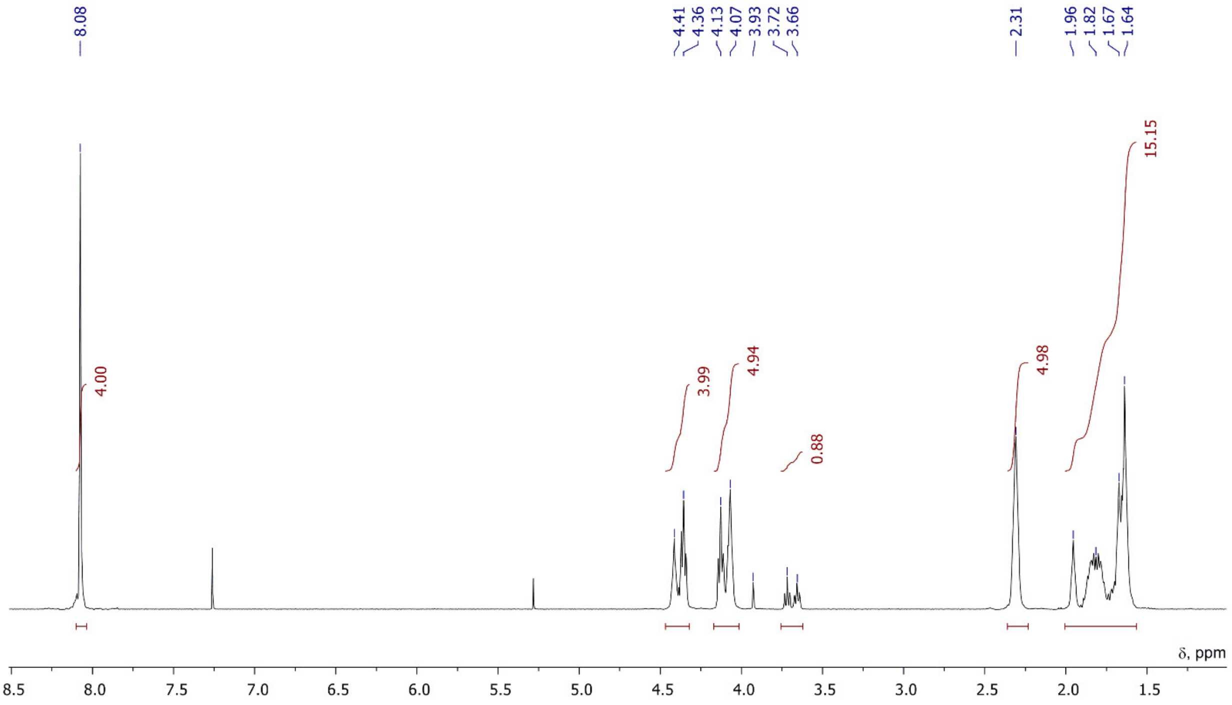

Figure S9. ^{1}H NMR spectrum (400 MHz, $CDCl_3$, 20 °C) of PBAT oligomer, obtained with the use of **4**.

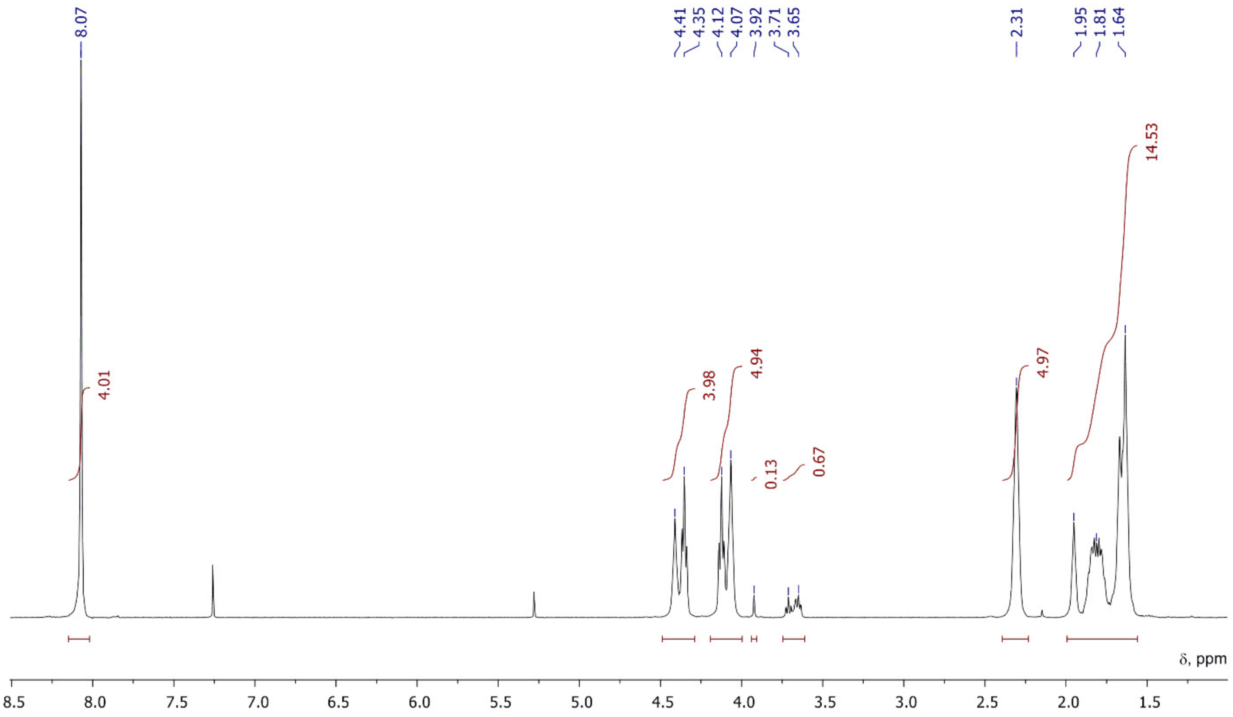

Figure S10. ^{1}H NMR spectrum (400 MHz, $CDCl_3$, 20 °C) of PBAT oligomer, obtained with the use of **5**.

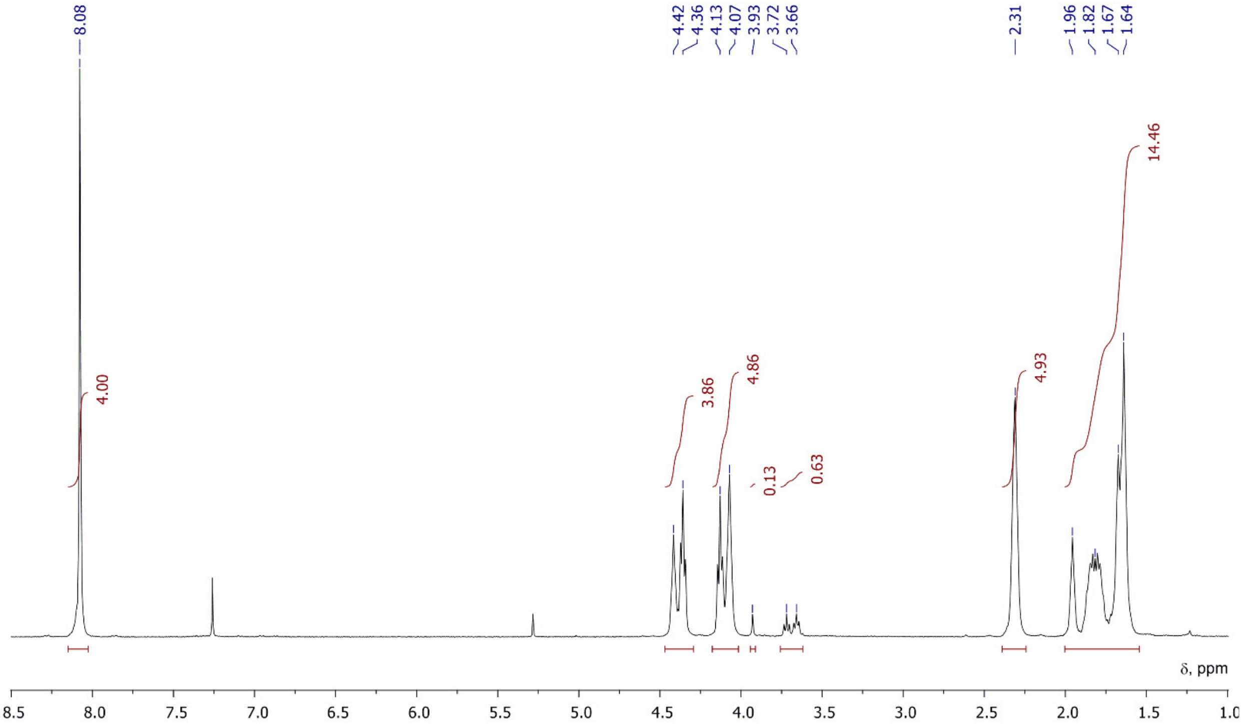

Figure S11. ^{1}H NMR spectrum (400 MHz, $CDCl_3$, 20 °C) of PBAT oligomer, obtained with the use of **6**.

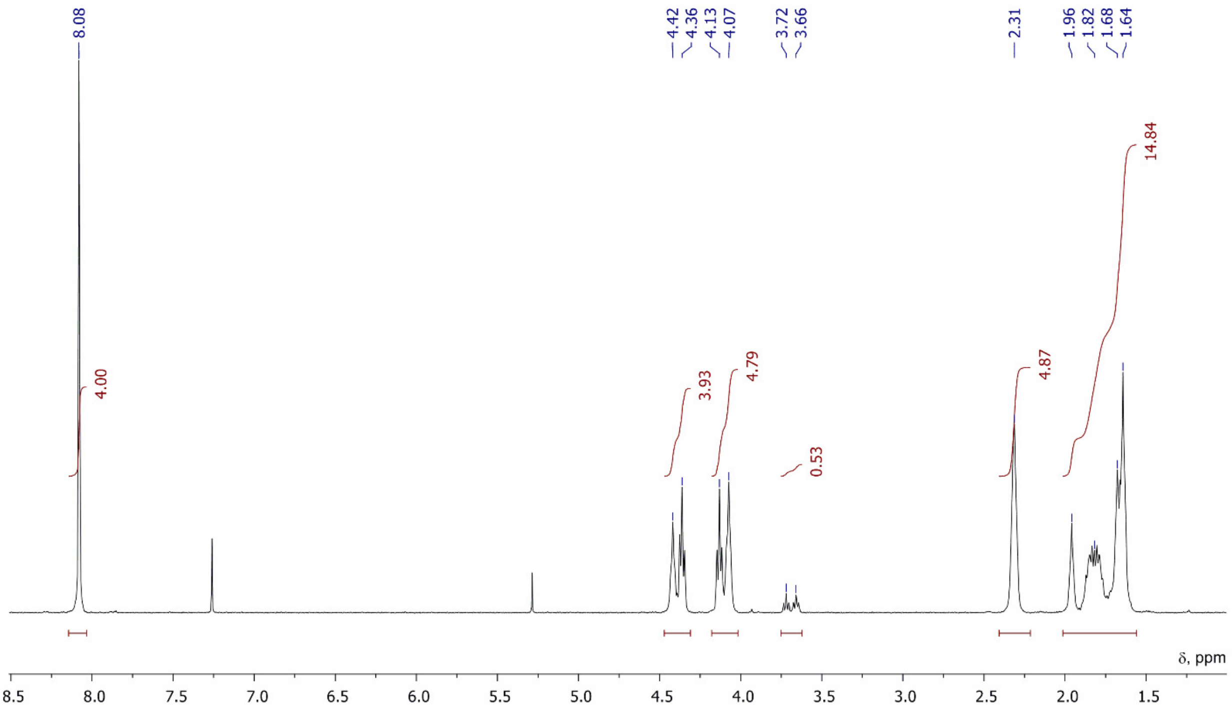

Figure S12. ^{1}H NMR spectrum (400 MHz, $CDCl_3$, 20 °C) of PBAT oligomer, obtained with the use of **7**.

S2.2. ^{1}H NMR spectra of the PBAT polymers (2nd stage)

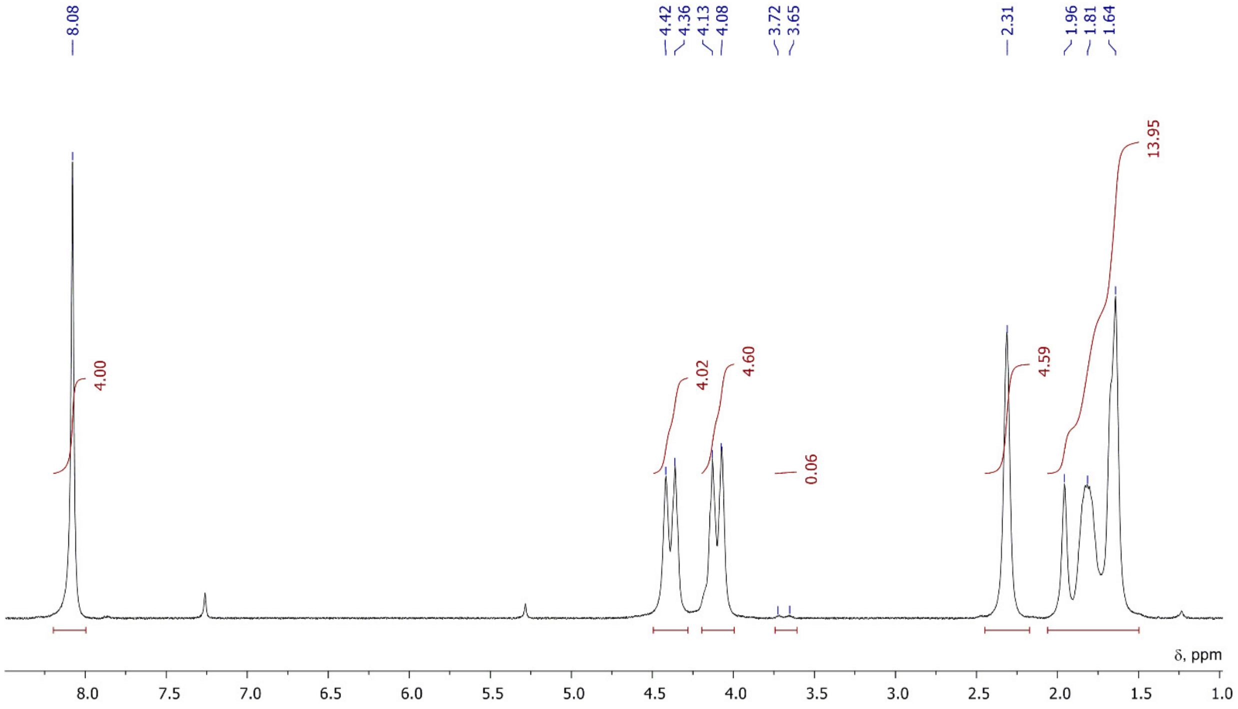

Figure S13. ^{1}H NMR spectrum (400 MHz, $CDCl_3$, 20 °C) of PBAT sample P1.

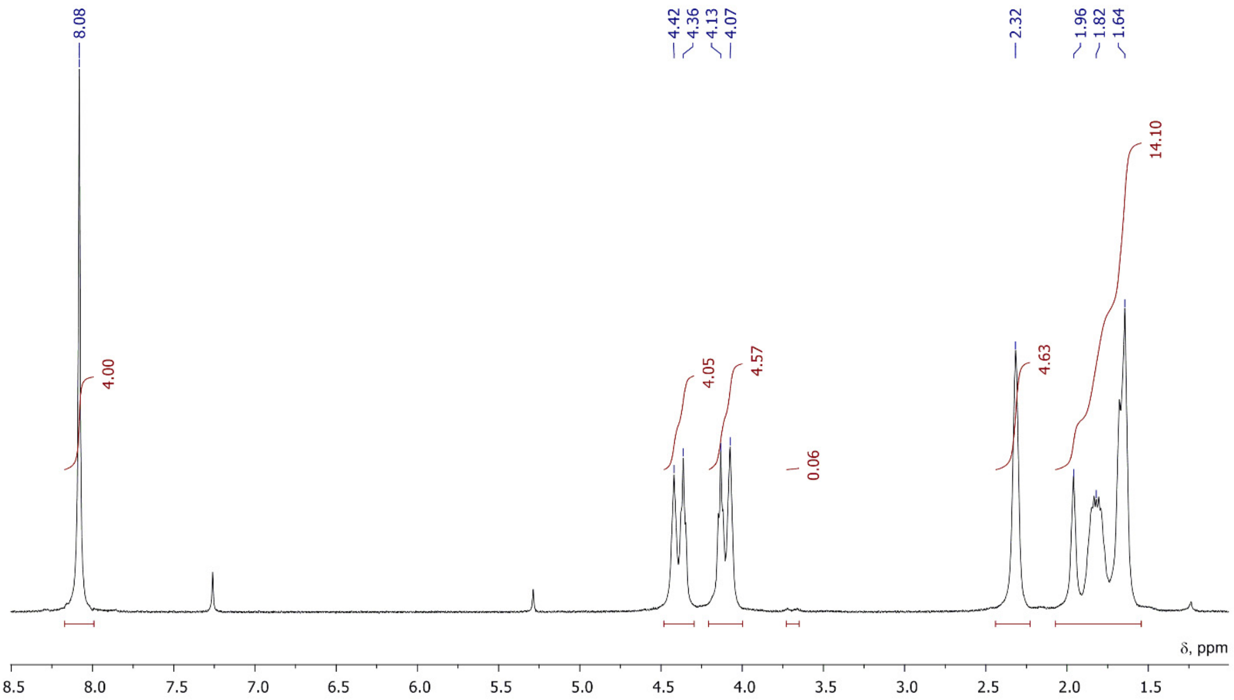

Figure S14. ^{1}H NMR spectrum (400 MHz, $CDCl_3$, 20 °C) of PBAT sample P2.

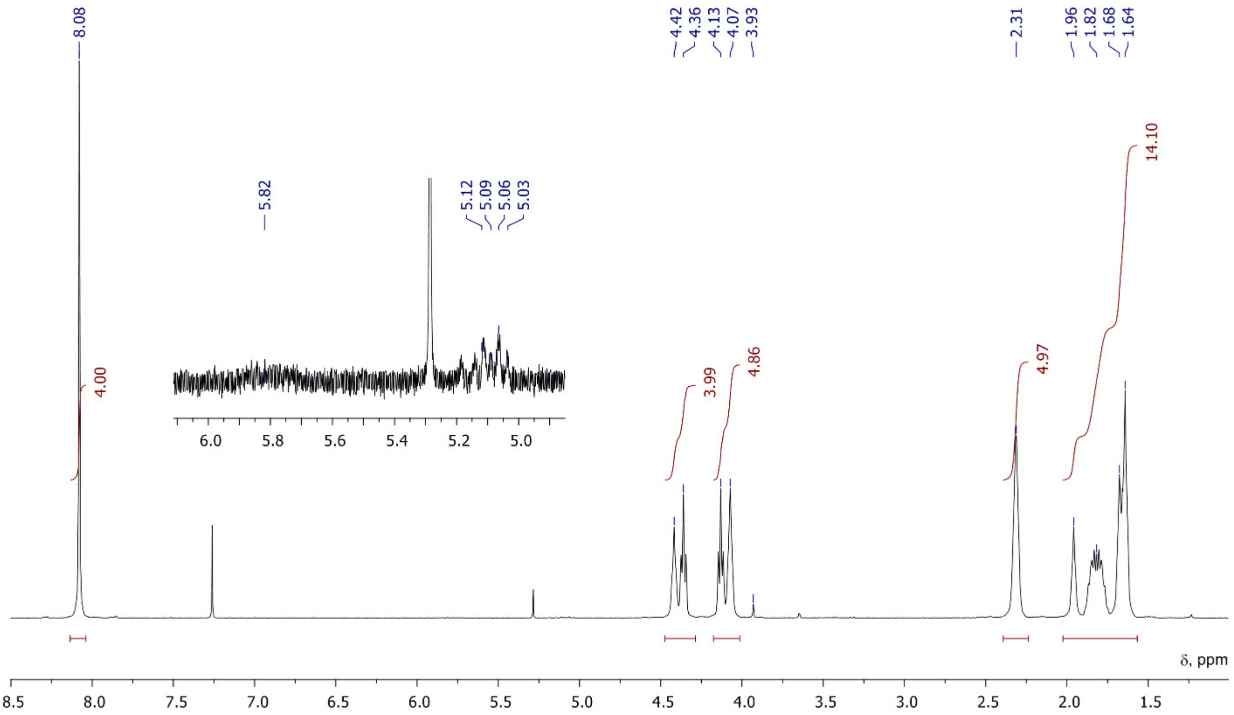

Figure S15. ^{1}H NMR spectrum (400 MHz, $CDCl_3$, 20 °C) of PBAT sample P3.

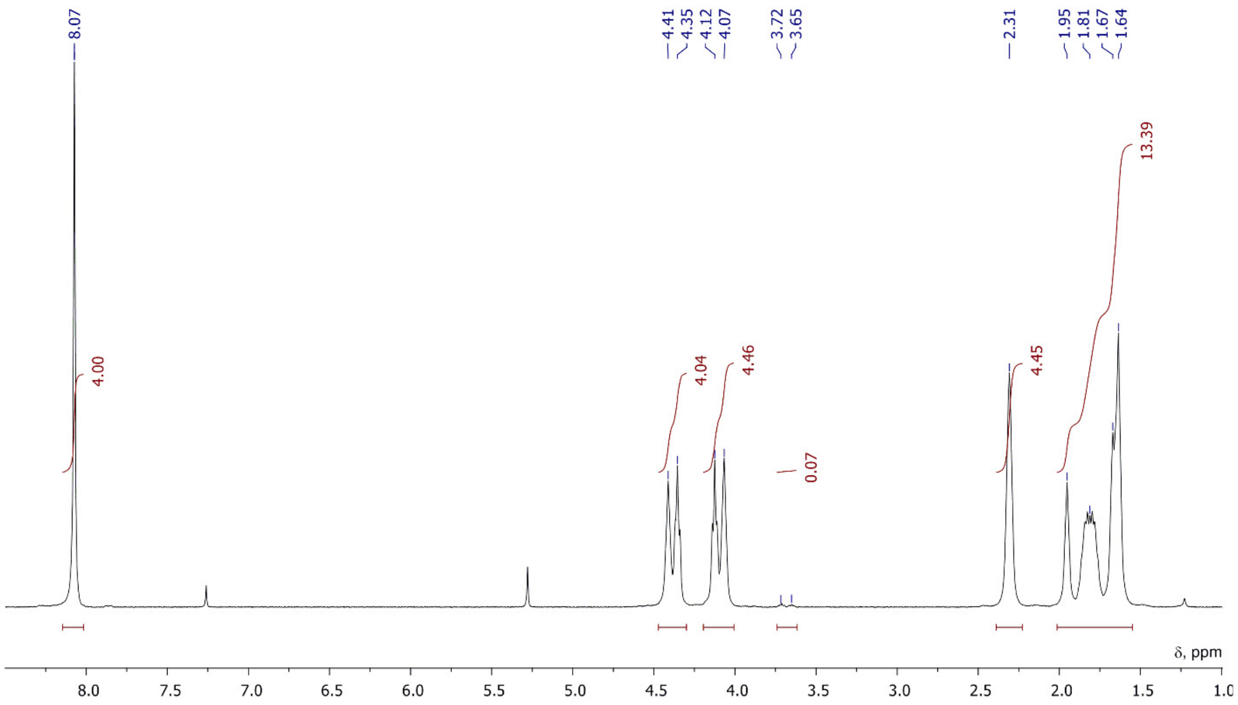

Figure S16. ^{1}H NMR spectrum (400 MHz, $CDCl_3$, 20 °C) of PBAT sample P4.

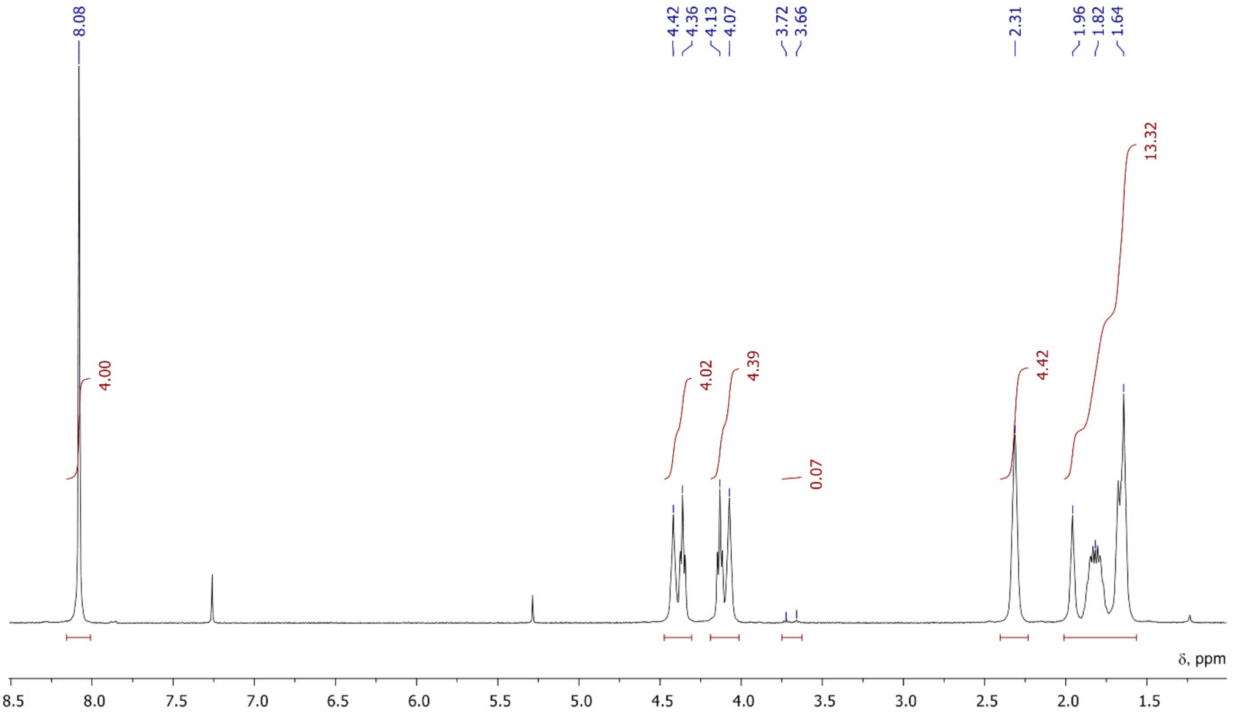

Figure S17. ^{1}H NMR spectrum (400 MHz, $CDCl_3$, 20 °C) of PBAT sample P5.

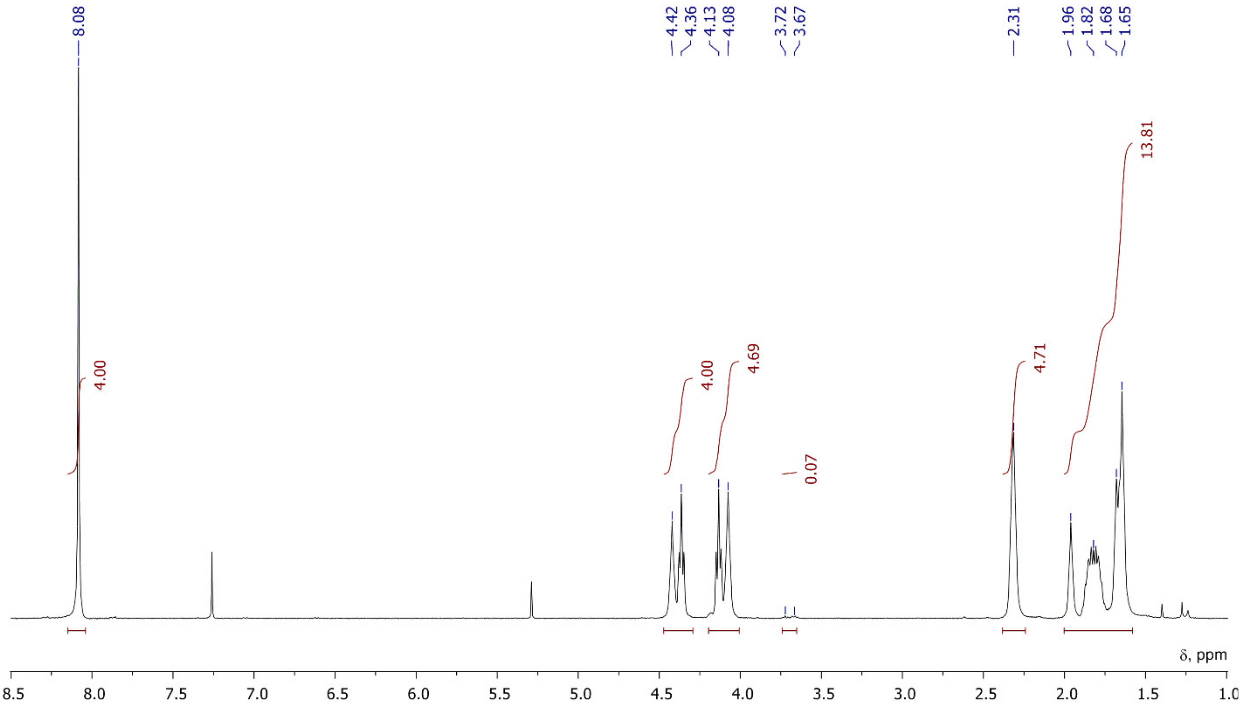

Figure S18. ^{1}H NMR spectrum (400 MHz, $CDCl_3$, 20 °C) of PBAT sample P6.

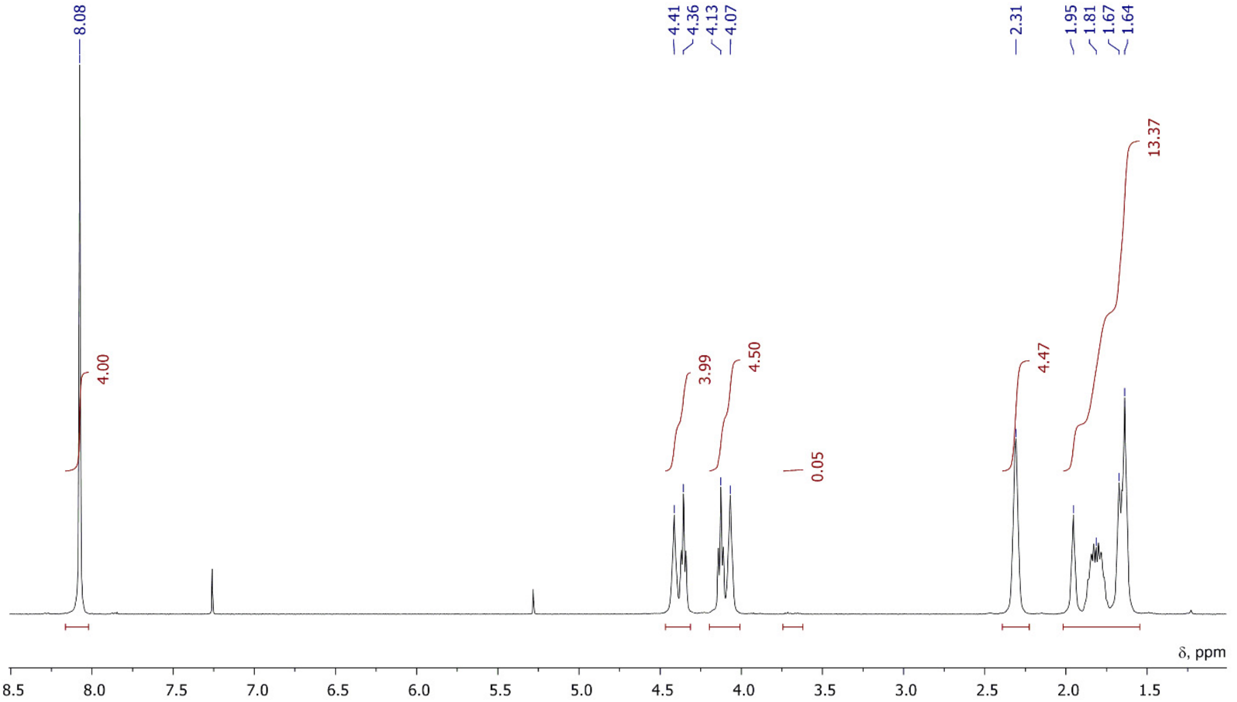

Figure S19. ^{1}H NMR spectrum (400 MHz, $CDCl_3$, 20 °C) of PBAT sample P7.

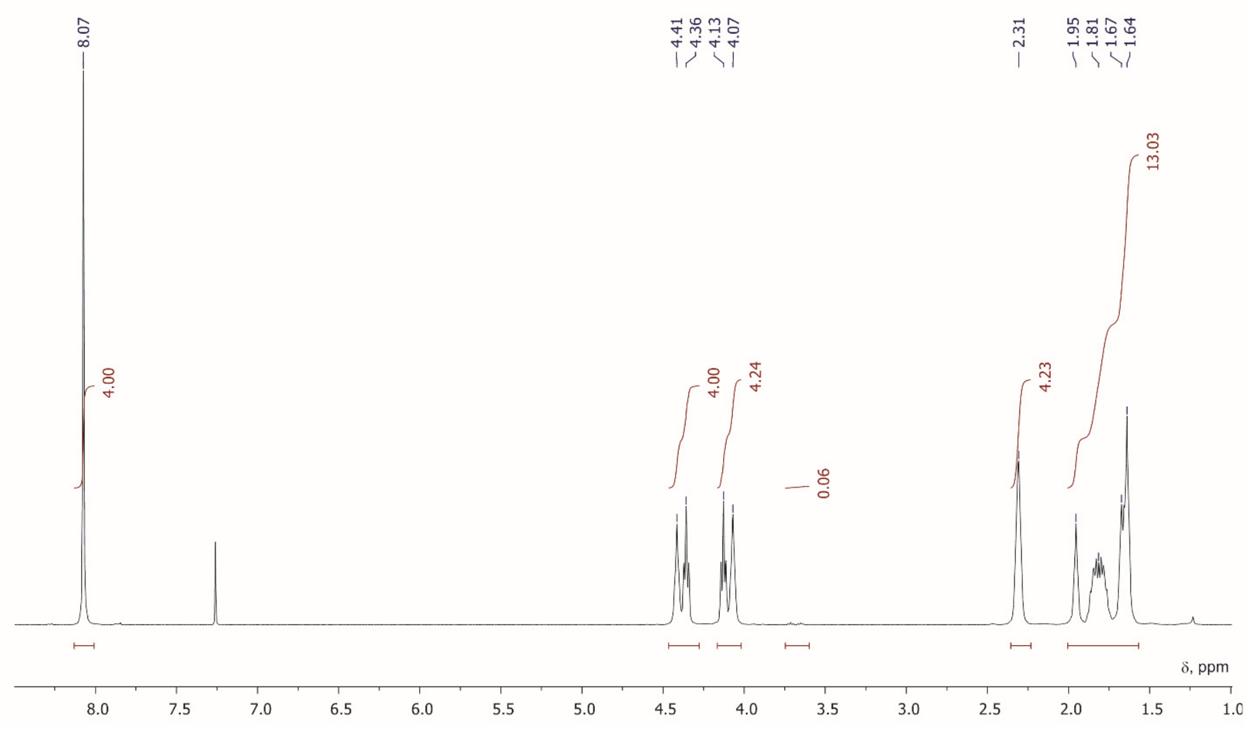

Figure S20. ^{1}H NMR spectrum (400 MHz, $CDCl_3$, 20 °C) of PBAT sample P8.

S2.3. ¹H and ¹³C NMR spectra of 8

Cyclic diester **8** represents colorless crystalline compound, obtained previously by the reaction of acipic acid with 1,4-butanediol without appropriate characterization [1]. Here we reproduce NMR spectra of **8**, first presented in our recent work [2].

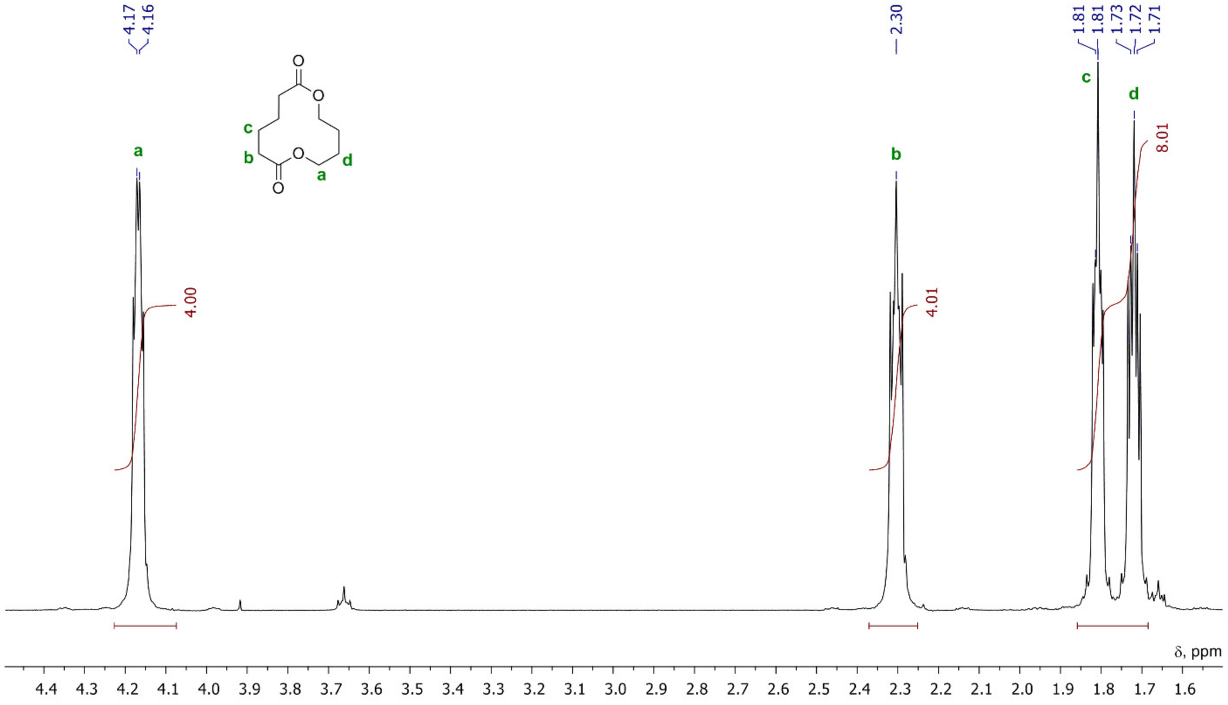

Figure S21. ¹H NMR spectrum (400 MHz, $CDCl_3$, 20 °C) of **8**.

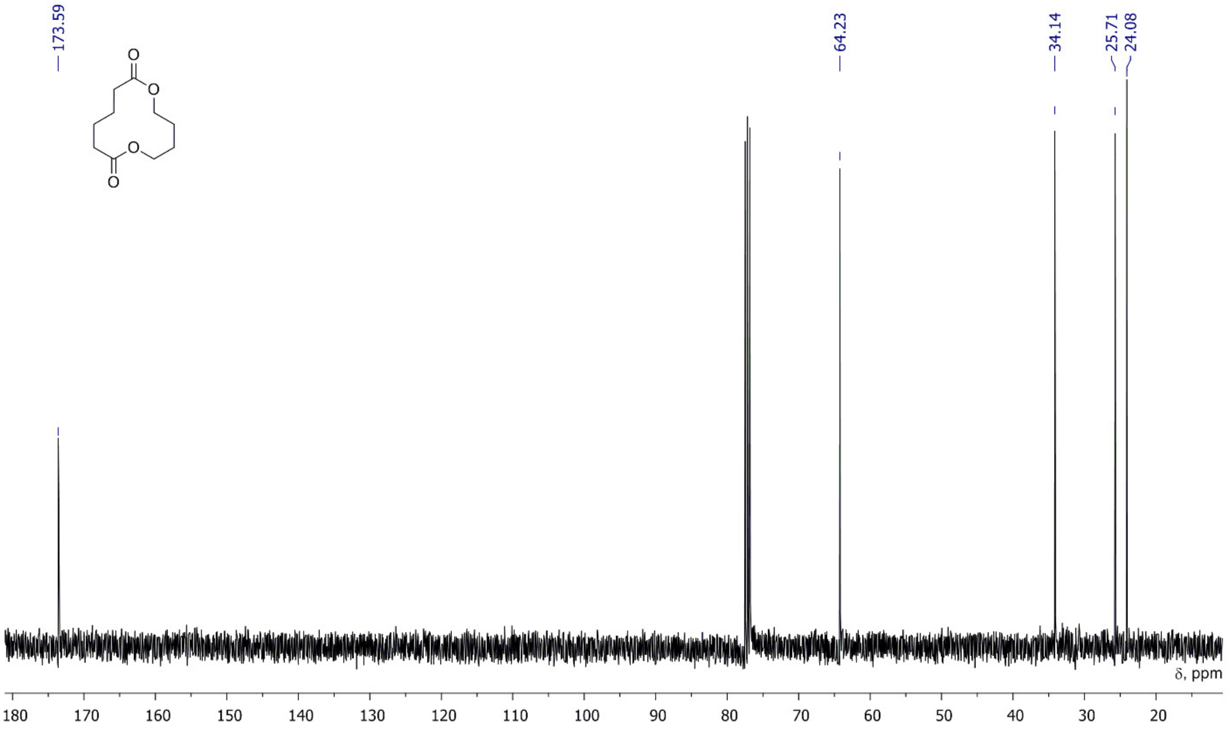

Figure S22. ¹³C {¹H} NMR spectrum (101 MHz, $CDCl_3$, 20 °C) of **8**.

S3. Thermal properties of PBATs

The thermal transition data for PBAT samples P1–P8 are summarized in Table S1. All samples crystallized readily from melt at a cooling rate of 5 °C/min. The absence of crystallization peaks in the second heating scan curves indicates that the melt crystallization was completed during cooling scans. The first and the second heating scans were similar for all samples under study (see Figs. S23–S30).

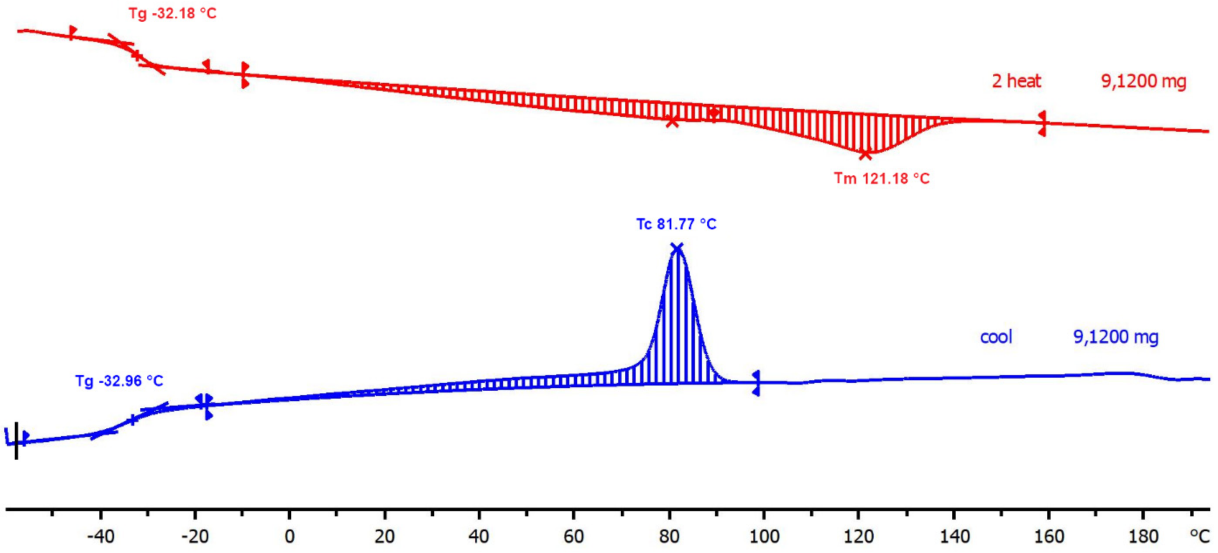

Figure S23. DSC plots of PBAT sample P1.

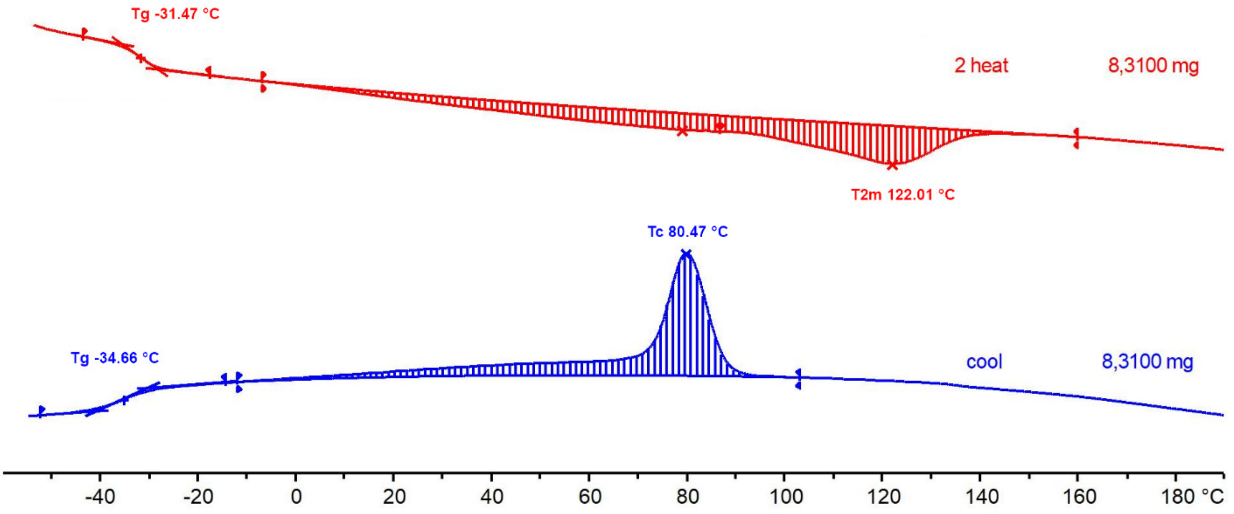

Figure S24. DSC plots of PBAT sample P2.

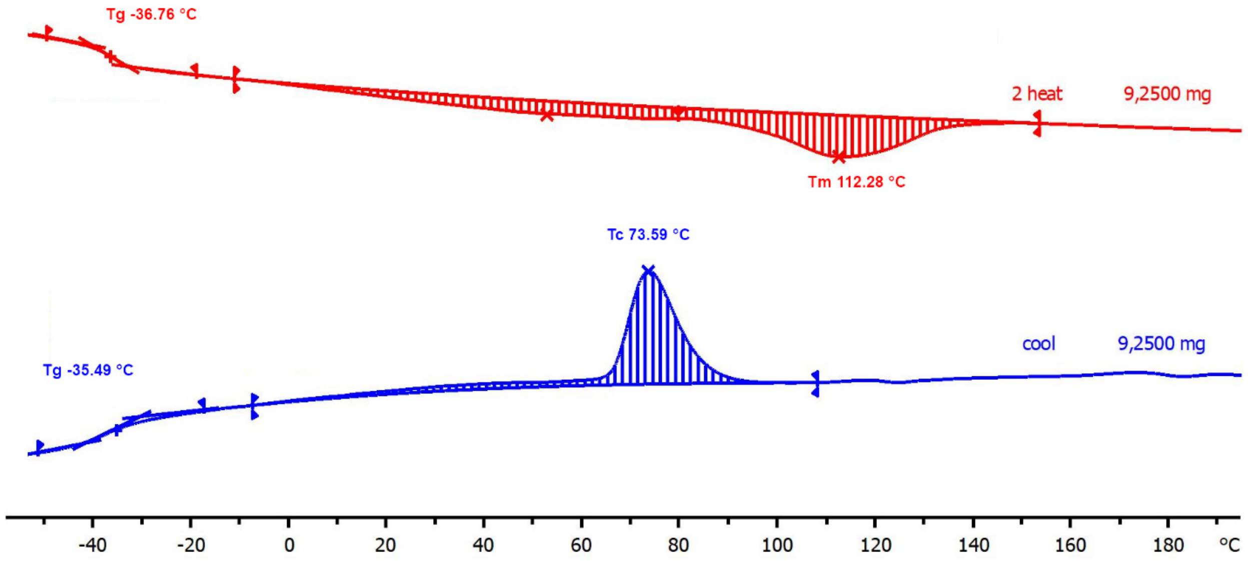

Figure S25. DSC plots of PBAT sample P3.

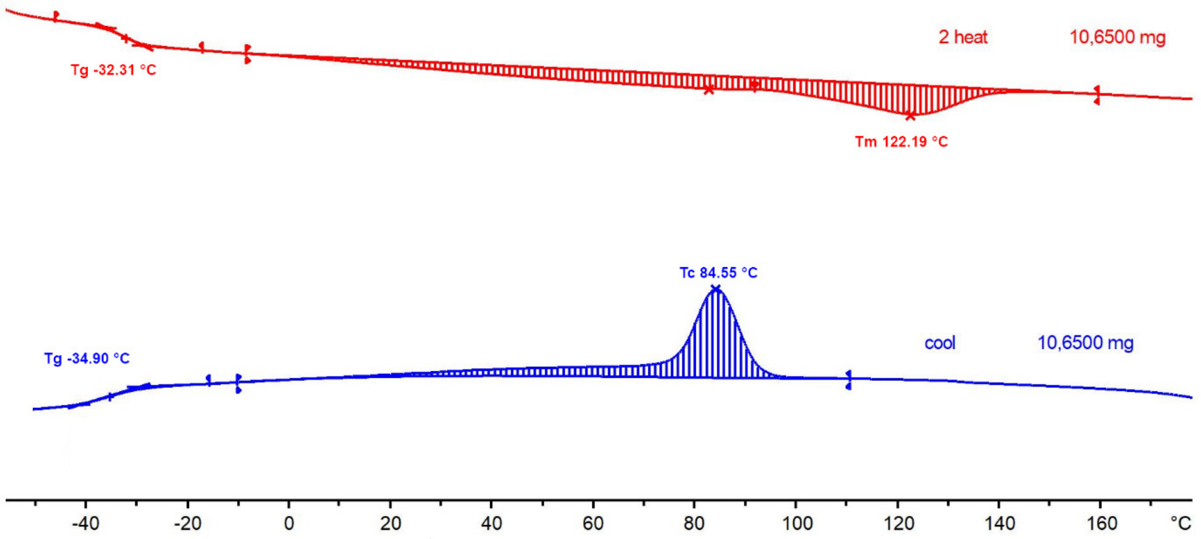

Figure S26. DSC plots of PBAT sample P4.

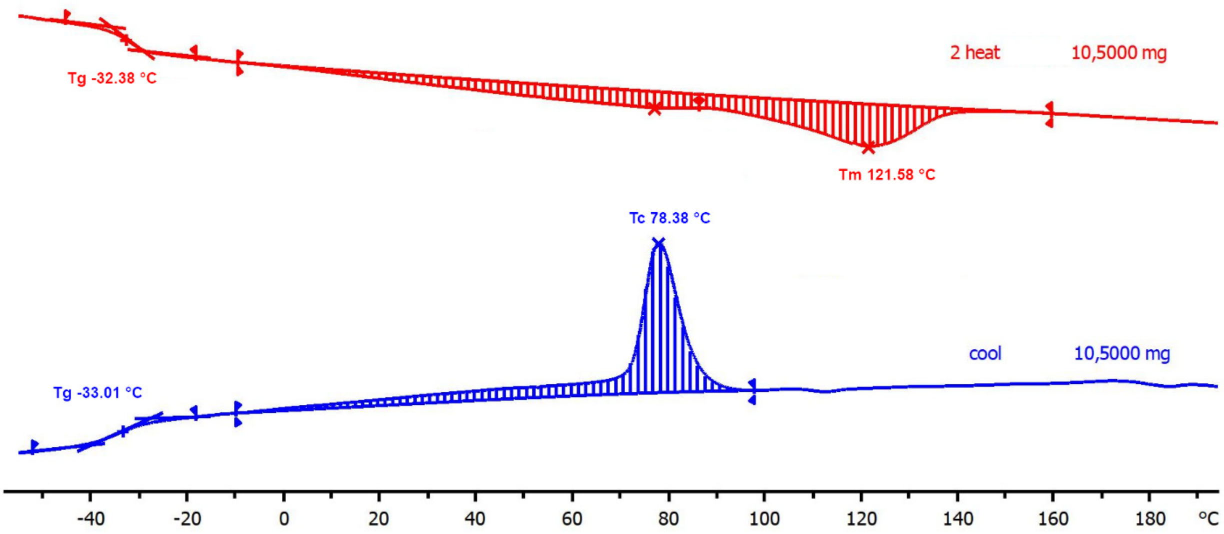

Figure S27. DSC plots of PBAT sample P5.

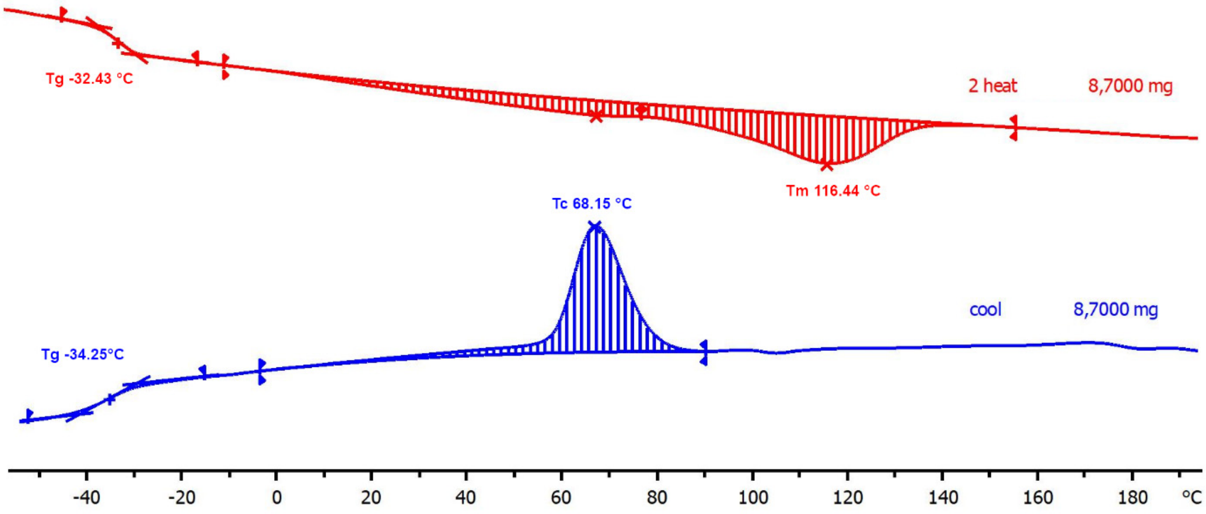

Figure S28. DSC plots of PBAT sample P6.

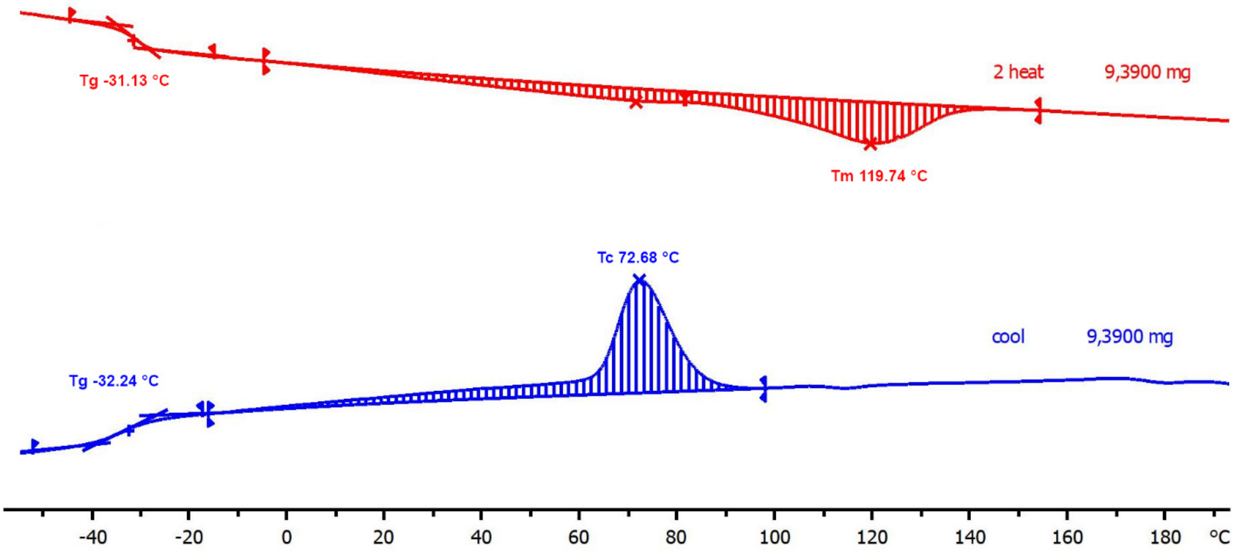

Figure S29. DSC plots of PBAT sample P7.

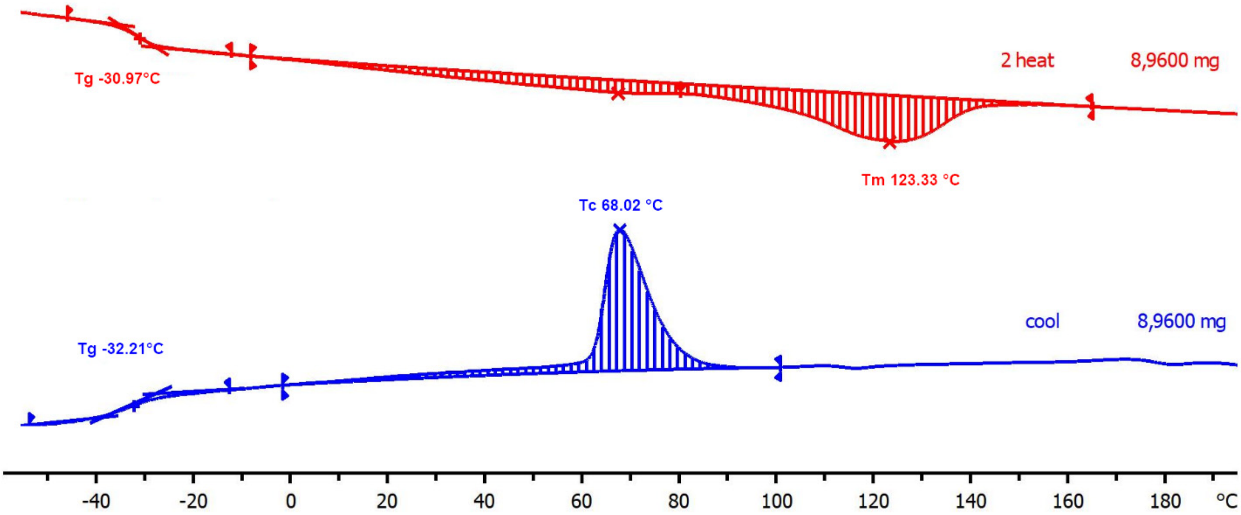

Figure S30. DSC plots of PBAT sample P8.

During the second heating scans, we observed broad melting peaks T_m at 112.3–123.3 °C that correspond to statistical PBAT copolymers [3,4]. The values of the T_m of PBAT are intermediate between T_m of poly(butylene adipate) (PBA, ~56 °C) and poly(butylene terephthalate) (PBT, ~227 °C) [3]. Note that both lower-temperature and higher-temperature melting peaks, that correspond to PBA and PBT blocks, respectively, were found to be virtually absent.

As the macromolecular chain becomes more branched, the glass transition temperature of the polymer increases [5]. At first glance, it seems that in our case the opposite is true: T_g decreases with increasing branching (Table S1). However, the glass transition temperature is affected by the ratio of monomer units in the copolymer, and can be estimated using the Fox equation (1) [6], which is a weighted harmonic mean of the glass transition temperatures of homopolymers [7].

$$\frac{1}{T_g} = \frac{w_1}{T_{g1}} + \frac{w_2}{T_{g2}} \quad (1)$$

where $T_{g,1}$ and $T_{g,2}$ are the glass transition temperatures of polymers from monomers 1 and 2, which have mass fractions w_1 and w_2 in the copolymer composition.

Table S1. Thermal characteristics of PBAT.

Entry	BA	2nd Heating scan			Cooling scan		
		T_g, °C	T_m, °C	ΔH_m, J/g	T_g, °C	T_c, °C	ΔH_c, J/g
P1	**1**	–32.18	121.18	-26.22	–32.96	81.77	22.06
P2	**2**	–31.47	122.01	-28.44	–34.66	80.47	25.26
P3	**3**	–36.76	112.28	-26.75	–35.49	73.59	20.36
P4	**4**	–32.31	122.19	-30.33	–34.90	84.55	24.52
P5	**5**	–32.38	121.58	-24.92	–33.01	78.38	22.29
P6	**6**	–32.43	116.44	-23.03	–34.25	68.15	18.60
P7	**7**	–31.13	119.74	-23.96	–32.24	72.68	26.09
P8	no	–30.97	123.33	-28.72	–32.21	68.02	22.80

The T_g of PBT and PBA are 24 °C [8] and –68 °C [9], respectively, which allows calculating the theoretical glass transition temperatures of copolymers (the line in Figure S31) based on A/T ratio (Table S1).

The glass transition temperature of linear PBAT (P8) lies on the calculated curve, while T_g of other samples are shifted upward relative to it. Higher glass transition temperatures for other samples can only be related to their branched nature. The greater the deviation from the theoretical T_g value, the higher the degree of branching. Thus, it can be supposed that the samples P2 and P7 are the most branched, which may be desirable in terms of obtaining the demanded polymer product.

According to the data presented in Table S1, the use of BAs substantially affect other thermal thermal characteristics of PBAT. The melting temperatures T_m decreased with branching. The values of melting enthalpy ΔH_m for branched PBAT decreased in comparison with linear PBAT, except copolymer sample P4 obtained in the presence of triester BA **4** (Table S1, Entry 4). After branching, the melt crystallization temperature T_c increased.

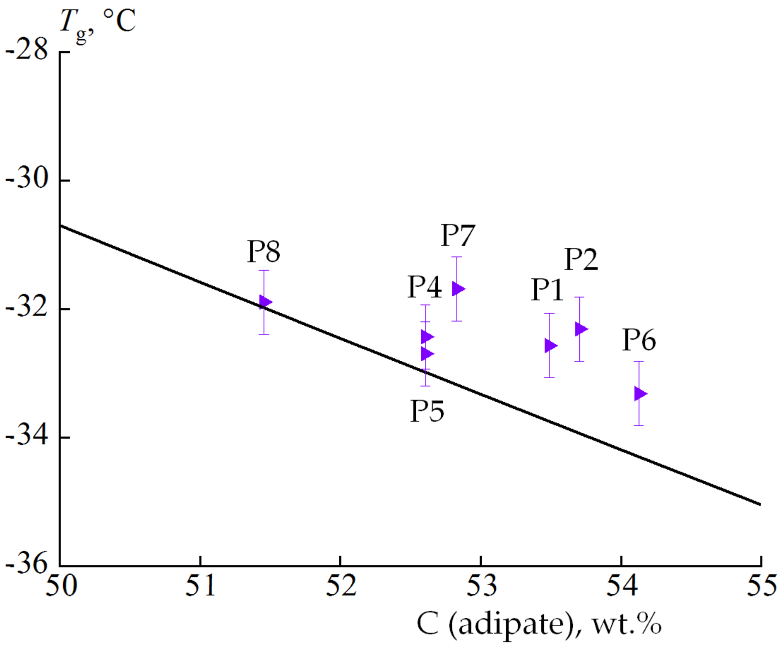

Figure S31. Dependence of the glass transition temperature of PBAT on the content of butylene adipate units. The dots show experimental data, while the line represents the result of a theoretical calculation using the Fox equation. The polymer numbers (see Table S1) are indicated near the dots. The data for low-MW copolymer P3 are not presented.

S4. Rheology of PBATs

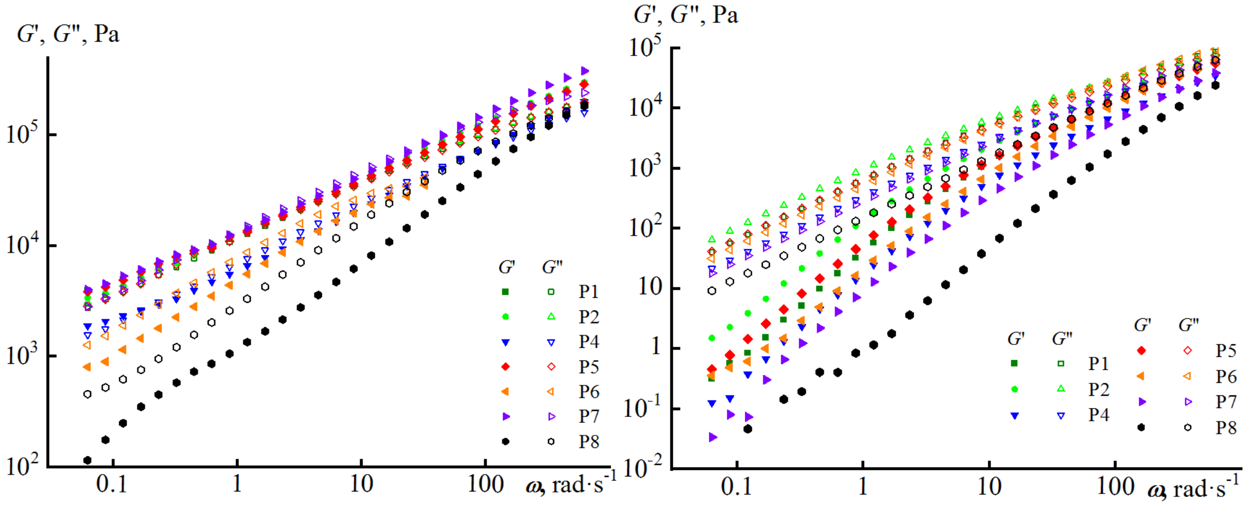

Figure S32. Frequency dependences of the storage and loss moduli at 130 °C (left) and 190 °C (right). The polymer numbers are presented in the legends.

References

1. Binns, F.; Taylor, A. Isolation, synthesis and reactivity of a key macrobislactone; 1,4,7-trioxacyclotridecane-8-13-dione. *Tetrahedron* **1995**, *51*, 12929–12938. DOI: 10.1016/0040-4020(95)00738-t.
2. Nifant'ev, I.E.; Bagrov, V.V.; Komarov, P.D.; Ovchinnikova, V.I.; Ivchenko, P.V. Aryloxy 'biometal' complexes as efficient catalysts for the synthesis of poly(butylene adipate terephthalate). *Mendeleev Commun.* **2022**, accepted. DOI: 10.1016/j.mencom.2022.05.020.
3. Shi, X.Q.; Ito, H.; Kikutani, T. Characterization on mixed-crystal structure and properties of poly(butylene adipate-co-terephthalate) biodegradable fibers. *Polymer* **2005**, *46*, 11442–11450. DOI: 10.1016/j.polymer.2005.10.065.
4. Chang, B.; Li, Y.; Wang, W.; Song, G.; Lin, J.; Murugadoss, V.; Naik, N.; Guo, Z. Impacts of chain extenders on thermal property, degradation, and rheological performance of poly(butylene adipate-co-terephthalate). *J. Mater. Res.* **2021**, *36*, 3134–3144. DOI: 10.1557/s43578-021-00308-0.
5. Askadskiĭ, A.A. *Computational Materials Science of Polymers*; Cambridge International Science Publishing: Cambridge, UK, 2003.
6. Hiemenz, P.C.; Lodge, T.P. *Polymer Chemistry*, 2nd ed.; CRC Press, Taylor & Franks Group: Boca Raton, USA, 2007.
7. Ilyin, S.O.; Makarova, V.V.; Polyakova, M.Y.; Kulichikhin, V.G. Phase state and rheology of polyisobutylene blends with silicone resin. *Rheol. Acta* **2020**, *59*, 375–386. DOI: 10.1007/s00397-020-01208-6.
8. Konishi, T.; Miyamoto, Y. Smectic structure and glass transition in poly(butylene terephthalate). *Polym. J.* **2010**, *42*, 349–353. DOI: 10.1038/pj.2010.5.
9. Schneider, N.S.; Sung, C.S.P. Transition behavior and phase segregation in TDI polyurethanes. *Polym. Eng. Sci.* **1977**, *17*, 73–80. DOI: 10.1002/pen.760170203.
